# Aggregation tests identify new gene associations with breast cancer in populations with diverse ancestry

**DOI:** 10.1186/s13073-022-01152-5

**Published:** 2023-01-26

**Authors:** Stefanie H. Mueller, Alvina G. Lai, Maria Valkovskaya, Kyriaki Michailidou, Manjeet K. Bolla, Qin Wang, Joe Dennis, Michael Lush, Zomoruda Abu-Ful, Thomas U. Ahearn, Irene L. Andrulis, Hoda Anton-Culver, Natalia N. Antonenkova, Volker Arndt, Kristan J. Aronson, Annelie Augustinsson, Thais Baert, Laura E. Beane Freeman, Matthias W. Beckmann, Sabine Behrens, Javier Benitez, Marina Bermisheva, Carl Blomqvist, Natalia V. Bogdanova, Stig E. Bojesen, Bernardo Bonanni, Hermann Brenner, Sara Y. Brucker, Saundra S. Buys, Jose E. Castelao, Tsun L. Chan, Jenny Chang-Claude, Stephen J. Chanock, Ji-Yeob Choi, Wendy K. Chung, NBCS Collaborators, Kristine K. Sahlberg, Anne-Lise Børresen-Dale, Lars Ottestad, Rolf Kåresen, Ellen Schlichting, Marit Muri Holmen, Toril Sauer, Vilde Haakensen, Olav Engebråten, Bjørn Naume, Alexander Fosså, Cecile E. Kiserud, Kristin V. Reinertsen, Åslaug Helland, Margit Riis, Jürgen Geisler, Grethe I. Grenaker Alnaes, Sarah V. Colonna, CTS Consortium, Sten Cornelissen, Fergus J. Couch, Kamila Czene, Mary B. Daly, Peter Devilee, Thilo Dörk, Laure Dossus, Miriam Dwek, Diana M. Eccles, Arif B. Ekici, A. Heather Eliassen, Christoph Engel, D. Gareth Evans, Peter A. Fasching, Olivia Fletcher, Henrik Flyger, Manuela Gago-Dominguez, Yu-Tang Gao, Montserrat García-Closas, José A. García-Sáenz, Jeanine Genkinger, Aleksandra Gentry-Maharaj, Felix Grassmann, Pascal Guénel, Melanie Gündert, Lothar Haeberle, Eric Hahnen, Christopher A. Haiman, Niclas Håkansson, Per Hall, Elaine F. Harkness, Patricia A. Harrington, Jaana M. Hartikainen, Mikael Hartman, Alexander Hein, Weang-Kee Ho, Maartje J. Hooning, Reiner Hoppe, John L. Hopper, Richard S. Houlston, Anthony Howell, David J. Hunter, Dezheng Huo, ABCTB Investigators, Deborah Marsh, Rodney Scott, Robert Baxter, Desmond Yip, Jane Carpenter, Alison Davis, Nirmala Pathmanathan, Peter Simpson, Dinny Graham, Mythily Sachchithananthan, Hidemi Ito, Motoki Iwasaki, Anna Jakubowska, Wolfgang Janni, Esther M. John, Michael E. Jones, Audrey Jung, Rudolf Kaaks, Daehee Kang, Elza K. Khusnutdinova, Sung-Won Kim, Cari M. Kitahara, Stella Koutros, Peter Kraft, Vessela N. Kristensen, Katerina Kubelka-Sabit, Allison W. Kurian, Ava Kwong, James V. Lacey, Diether Lambrechts, Loic Le Marchand, Jingmei Li, Martha Linet, Wing-Yee Lo, Jirong Long, Artitaya Lophatananon, Arto Mannermaa, Mehdi Manoochehri, Sara Margolin, Keitaro Matsuo, Dimitrios Mavroudis, Usha Menon, Kenneth Muir, Rachel A. Murphy, Heli Nevanlinna, William G. Newman, Dieter Niederacher, Katie M. O’Brien, Nadia Obi, Kenneth Offit, Olufunmilayo I. Olopade, Andrew F. Olshan, Håkan Olsson, Sue K. Park, Alpa V. Patel, Achal Patel, Charles M. Perou, Julian Peto, Paul D. P. Pharoah, Dijana Plaseska-Karanfilska, Nadege Presneau, Brigitte Rack, Paolo Radice, Dhanya Ramachandran, Muhammad U. Rashid, Gad Rennert, Atocha Romero, Kathryn J. Ruddy, Matthias Ruebner, Emmanouil Saloustros, Dale P. Sandler, Elinor J. Sawyer, Marjanka K. Schmidt, Rita K. Schmutzler, Michael O. Schneider, Christopher Scott, Mitul Shah, Priyanka Sharma, Chen-Yang Shen, Xiao-Ou Shu, Jacques Simard, Harald Surowy, Rulla M. Tamimi, William J. Tapper, Jack A. Taylor, Soo Hwang Teo, Lauren R. Teras, Amanda E. Toland, Rob A. E. M. Tollenaar, Diana Torres, Gabriela Torres-Mejía, Melissa A. Troester, Thérèse Truong, Celine M. Vachon, Joseph Vijai, Clarice R. Weinberg, Camilla Wendt, Robert Winqvist, Alicja Wolk, Anna H. Wu, Taiki Yamaji, Xiaohong R. Yang, Jyh-Cherng Yu, Wei Zheng, Argyrios Ziogas, Elad Ziv, Alison M. Dunning, Douglas F. Easton, Harry Hemingway, Ute Hamann, Karoline B. Kuchenbaecker

**Affiliations:** 1 Institute of Health Informatics, University College London, London, UK; 2 Division of Psychiatry, University College London, London, UK; 3 Biostatistics Unit, The Cyprus Institute of Neurology and Genetics, 2371 Nicosia, Cyprus; 4 Cyprus School of Molecular Medicine, The Cyprus Institute of Neurology and Genetics, 2371 Nicosia, Cyprus; 5 Centre for Cancer Genetic Epidemiology, Department of Public Health and Primary Care, University of Cambridge, Cambridge, CB1 8RN UK; 6 Clalit National Cancer Control Center, Carmel Medical Center and Technion Faculty of Medicine, 35254 Haifa, Israel; 7 Division of Cancer Epidemiology and Genetics, Department of Health and Human Services, National Cancer Institute, National Institutes of Health, Bethesda, MD 20850 USA; 8 Fred A. Litwin Center for Cancer Genetics, Lunenfeld-Tanenbaum Research Institute of Mount Sinai Hospital, Toronto, ON M5G 1X5 Canada; 9 Department of Molecular Genetics, University of Toronto, Toronto, ON M5S 1A8 Canada; 10 Department of Medicine, Genetic Epidemiology Research Institute, University of California Irvine, Irvine, CA 92617 USA; 11 N.N. Alexandrov Research Institute of Oncology and Medical Radiology, 223040 Minsk, Belarus; 12 Division of Clinical Epidemiology and Aging Research, German Cancer Research Center (DKFZ), 69120 Heidelberg, Germany; 13 Department of Public Health Sciences, and Cancer Research Institute, Queen’s University, Kingston, ON K7L 3N6 Canada; 14 Department of Cancer Epidemiology, Clinical Sciences, Lund University, 222 42 Lund, Sweden; 15 Leuven Multidisciplinary Breast Center, Department of Oncology, Leuven Cancer Institute, University Hospitals Leuven, 3000 Louvain, Belgium; 16 Department of Gynecology and Obstetrics, Comprehensive Cancer Center Erlangen-EMN, University Hospital Erlangen, Friedrich-Alexander University Erlangen-Nuremberg (FAU), 91054 Erlangen, Germany; 17 Division of Cancer Epidemiology, German Cancer Research Center (DKFZ), 69120 Heidelberg, Germany; 18 Biomedical Network On Rare Diseases (CIBERER), 28029 Madrid, Spain; 19 Human Cancer Genetics Programme, Spanish National Cancer Research Centre (CNIO), 28029 Madrid, Spain; 20 Institute of Biochemistry and Genetics, Ufa Federal Research Centre of the Russian Academy of Sciences, Ufa, 450054 Russia; 21 Department of Oncology, Helsinki University Hospital, University of Helsinki, 00290 Helsinki, Finland; 22 Department of Oncology, Örebro University Hospital, 70185 Örebro, Sweden; 23 Department of Radiation Oncology, Hannover Medical School, 30625 Hannover, Germany; 24 Gynaecology Research Unit, Hannover Medical School, 30625 Hannover, Germany; 25 Copenhagen General Population Study, Herlev and Gentofte Hospital, Copenhagen University Hospital, 2730 Herlev, Denmark; 26 Department of Clinical Biochemistry, Herlev and Gentofte Hospital, Copenhagen University Hospital, 2730 Herlev, Denmark; 27 Faculty of Health and Medical Sciences, University of Copenhagen, 2200 Copenhagen, Denmark; 28 Division of Cancer Prevention and Genetics, IEO, European Institute of Oncology IRCCS, 20141 Milan, Italy; 29 Division of Preventive Oncology, German Cancer Research Center (DKFZ), National Center for Tumor Diseases (NCT), 69120 Heidelberg, Germany; 30 German Cancer Consortium (DKTK), German Cancer Research Center (DKFZ), 69120 Heidelberg, Germany; 31 Department of Gynecology and Obstetrics, University of Tübingen, 72076 Tübingen, Germany; 32 Department of Medicine, Huntsman Cancer Institute, Salt Lake City, UT 84112 USA; 33 Oncology and Genetics Unit, Instituto de Investigación Sanitaria Galicia Sur (IISGS), Xerencia de Xestion Integrada de Vigo-SERGAS, 36312 Vigo, Spain; 34 Hong Kong Hereditary Breast Cancer Family Registry, Hong Kong, China; 35 Department of Molecular Pathology, Hong Kong Sanatorium and Hospital, Hong Kong, China; 36 Cancer Epidemiology Group, University Cancer Center Hamburg (UCCH), University Medical Center Hamburg-Eppendorf, 20246 Hamburg, Germany; 37 Department of Biomedical Sciences, Seoul National University Graduate School, Seoul, 03080 Korea; 38 Cancer Research Institute, Seoul National University, Seoul, 03080 Korea; 39 Institute of Health Policy and Management, Seoul National University Medical Research Center, Seoul, 03080 Korea; 40 Departments of Pediatrics and Medicine, Columbia University, New York, NY 10032 USA; 41 Department of Cancer Genetics, Institute for Cancer Research, Oslo University Hospital-Radiumhospitalet, 0379 Oslo, Norway; 42 Institute of Clinical Medicine, Faculty of Medicine, University of Oslo, 0450 Oslo, Norway; 43 Department of Research, Vestre Viken Hospital, 3019 Drammen, Norway; 44 Section for Breast- and Endocrine Surgery, Department of Cancer, Division of Surgery, Cancer and Transplantation Medicine, Oslo University Hospital-Ullevål, 0450 Oslo, Norway; 45 Department of Radiology and Nuclear Medicine, Oslo University Hospital, 0379 Oslo, Norway; 46 Department of Pathology, Akershus University Hospital, 1478 Lørenskog, Norway; 47 Department of Tumor Biology, Institute for Cancer Research, Oslo University Hospital, 0379 Oslo, Norway; 48 Department of Oncology, Division of Surgery, Cancer and Transplantation Medicine, Oslo University Hospital-Radiumhospitalet, 0379 Oslo, Norway; 49 National Advisory Unit On Late Effects After Cancer Treatment, Oslo University Hospital, 0379 Oslo, Norway; 50 Department of Oncology, Akershus University Hospital, 1478 Lørenskog, Norway; 51 Oslo Breast Cancer Research Consortium, Oslo University Hospital, 0379 Oslo, Norway; 52 Department of Medical Genetics, Oslo University Hospital and University of Oslo, 0379 Oslo, Norway; 53 Department of Computational and Quantitative Medicine, City of Hope, Duarte, CA 91010 USA; 54 City of Hope Comprehensive Cancer Center, City of Hope, Duarte, CA 91010 USA; 55 Division of Molecular Pathology, The Netherlands Cancer Institute - Antoni Van Leeuwenhoek Hospital, Amsterdam, 1066 CX The Netherlands; 56 Department of Laboratory Medicine and Pathology, Mayo Clinic, Rochester, MN 55905 USA; 57 Department of Medical Epidemiology and Biostatistics, Karolinska Institutet, 171 65 Stockholm, Sweden; 58 Department of Clinical Genetics, Fox Chase Cancer Center, Philadelphia, PA 19111 USA; 59 Department of Pathology, Leiden University Medical Center, Leiden, 2333 ZA The Netherlands; 60 Department of Human Genetics, Leiden University Medical Center, Leiden, 2333 ZA The Netherlands; 61 Nutrition and Metabolism Section, International Agency for Research On Cancer (IARC-WHO), 69372 Lyon, France; 62 School of Life Sciences, University of Westminster, London, W1W 6UW UK; 63 Faculty of Medicine, University of Southampton, Southampton, SO17 1BJ UK; 64 Institute of Human Genetics, Comprehensive Cancer Center Erlangen-EMN, University Hospital Erlangen, Friedrich-Alexander University Erlangen-Nuremberg (FAU), 91054 Erlangen, Germany; 65 Channing Division of Network Medicine, Department of Medicine, Brigham and Women’s Hospital, Harvard Medical School, Boston, MA 02115 USA; 66 Department of Epidemiology, Harvard T.H. Chan School of Public Health, Boston, MA 02115 USA; 67 Department of Nutrition, Harvard T.H. Chan School of Public Health, Boston, MA 02115 USA; 68 Institute for Medical Informatics, Statistics and Epidemiology, University of Leipzig, 04107 Leipzig, Germany; 69 LIFE - Leipzig Research Centre for Civilization Diseases, University of Leipzig, 04103 Leipzig, Germany; 70 Division of Evolution and Genomic Sciences, School of Biological Sciences, Faculty of Biology, Medicine and Health, University of Manchester, Manchester Academic Health Science Centre, Manchester, M13 9WL UK; 71 North West Genomics Laboratory Hub, Manchester Centre for Genomic Medicine, St Mary’s Hospital, Manchester University NHS Foundation Trust, Manchester Academic Health Science Centre, Manchester, M13 9WL UK; 72 David Geffen School of Medicine, Department of Medicine Division of Hematology and Oncology, University of California at Los Angeles, Los Angeles, CA 90095 USA; 73 The Breast Cancer Now Toby Robins Research Centre, The Institute of Cancer Research, London, SW7 3RP UK; 74 Department of Breast Surgery, Herlev and Gentofte Hospital, Copenhagen University Hospital, 2730 Herlev, Denmark; 75 Genomic Medicine Group, International Cancer Genetics and Epidemiology Group, Fundación Pœblica Galega de Medicina Xenómica, Instituto de Investigación Sanitaria de Santiago de Compostela (IDIS), Complejo Hospitalario Universitario de Santiago, SERGAS, 15706 Santiago de Compostela, Spain; 76 Moores Cancer Center, University of California San Diego, La Jolla, CA 92037 USA; 77 Department of Epidemiology, Shanghai Cancer Institute, Shanghai, 20032 China; 78 Medical Oncology Department, Centro Investigación Biomédica en Red de Cáncer (CIBERONC), Hospital Clínico San Carlos, Instituto de Investigación Sanitaria San Carlos (IdISSC), 28040 Madrid, Spain; 79 Department of Epidemiology, Mailman School of Public Health, Columbia University, New York, NY 10032 USA; 80 Institute of Clinical Trials and Methodology, University College London, London, WC1V 6LJ UK; 81 Health and Medical University, 14471 Potsdam, Germany; 82 Center for Research in Epidemiology and Population Health (CESP), Team Exposome and Heredity, INSERM, University Paris-Saclay, 94805 Villejuif, France; 83 Molecular Epidemiology Group, German Cancer Research Center (DKFZ), C08069120 Heidelberg, Germany; 84 Molecular Biology of Breast Cancer, University Womens Clinic Heidelberg, University of Heidelberg, 69120 Heidelberg, Germany; 85 Institute of Diabetes Research, Helmholtz Zentrum München, German Research Center for Environmental Health, 85764 Neuherberg, Germany; 86 Center for Familial Breast and Ovarian Cancer, Faculty of Medicine, University Hospital Cologne, University of Cologne, 50937 Cologne, Germany; 87 Center for Integrated Oncology (CIO), Faculty of Medicine, University Hospital Cologne, University of Cologne, 50937 Cologne, Germany; 88 Department of Preventive Medicine, Keck School of Medicine, University of Southern California, Los Angeles, CA 90033 USA; 89 Institute of Environmental Medicine, Karolinska Institutet, 171 77 Stockholm, Sweden; 90 Department of Oncology, 118 83 Sšdersjukhuset, Stockholm, Sweden; 91 Division of Informatics, Imaging and Data Sciences, Faculty of Biology, Medicine and Health, University of Manchester, Manchester Academic Health Science Centre, Manchester, M13 9PT UK; 92 Nightingale and Genesis Prevention Centre, Wythenshawe Hospital, Manchester University NHS Foundation Trust, Manchester, M23 9LT UK; 93 NIHR Manchester Biomedical Research Unit, Manchester University NHS Foundation Trust, Manchester Academic Health Science Centre, Manchester, M13 9WL UK; 94 Centre for Cancer Genetic Epidemiology, Department of Oncology, University of Cambridge, Cambridge, CB1 8RN UK; 95 Translational Cancer Research Area, University of Eastern Finland, 70210 Kuopio, Finland; 96 Institute of Clinical Medicine, Pathology and Forensic Medicine, University of Eastern Finland, 70210 Kuopio, Finland; 97 Saw Swee Hock School of Public Health, National University of Singapore, National University Health System, Singapore, 119077 Singapore; 98 Department of Surgery, National University Health System, Singapore, 119228 Singapore; 99 Department of Mathematical Sciences, Faculty of Science and Engineering, University of Nottingham Malaysia Campus, 43500 Semenyih, Selangor Malaysia; 100 Breast Cancer Research Programme, Cancer Research Malaysia, Subang Jaya, 47500 Selangor, Malaysia; 101 Department of Medical Oncology, Erasmus MC Cancer Institute, Rotterdam, 3015 GD The Netherlands; 102 Dr. Margarete Fischer-Bosch-Institute of Clinical Pharmacology, 70376 Stuttgart, Germany; 103 University of Tübingen, 72074 Tübingen, Germany; 104 Centre for Epidemiology and Biostatistics, Melbourne School of Population and Global Health, The University of Melbourne, Melbourne, VIC 3010 Australia; 105 Division of Genetics and Epidemiology, The Institute of Cancer Research, London, SM2 5NG UK; 106 Division of Cancer Sciences, University of Manchester, Manchester, M13 9PL UK; 107 Nuffield Department of Population Health, University of Oxford, Oxford, OX3 7LF UK; 108 Center for Clinical Cancer Genetics, The University of Chicago, Chicago, IL 60637 USA; 109 Australian Breast Cancer Tissue Bank, Westmead Institute for Medical Research, University of Sydney, Sydney, NSW 2145 Australia; 110 Division of Cancer Information and Control, Aichi Cancer Center Research Institute, Nagoya, 464-8681 Japan; 111 Division of Cancer Epidemiology, Nagoya University Graduate School of Medicine, Nagoya, 466-8550 Japan; 112 Division of Epidemiology, Center for Public Health Sciences, National Cancer Center Institute for Cancer Control, Tokyo, 104-0045 Japan; 113 Department of Genetics and Pathology, Pomeranian Medical University, 71-252 Szczecin, Poland; 114 Independent Laboratory of Molecular Biology and Genetic Diagnostics, Pomeranian Medical University, 71-252 Szczecin, Poland; 115 Department of Gynaecology and Obstetrics, University Hospital Ulm, 89075 Ulm, Germany; 116 Department of Epidemiology and Population Health, Stanford University School of Medicine, Stanford, CA 94305 USA; 117 Department of Medicine, Division of Oncology, Stanford Cancer Institute, Stanford University School of Medicine, Stanford, CA 94304 USA; 118 Department of Preventive Medicine, Seoul National University College of Medicine, Seoul, 03080 Korea; 119 Department of Genetics and Fundamental Medicine, Bashkir State University, Ufa, 450000 Russia; 120 Department of Surgery, Daerim Saint Mary’s Hospital, Seoul, 07442 Korea; 121 Radiation Epidemiology Branch, Division of Cancer Epidemiology and Genetics, National Cancer Institute, Bethesda, MD 20892 USA; 122 Program in Genetic Epidemiology and Statistical Genetics, Harvard T.H. Chan School of Public Health, Boston, MA 02115 USA; 123 Department of Histopathology and Cytology, Clinical Hospital Acibadem Sistina, Skopje, 1000 Republic of North Macedonia; 124 Department of Surgery, The University of Hong Kong, Hong Kong, China; 125 Department of Surgery and Cancer Genetics Center, Hong Kong Sanatorium and Hospital, Hong Kong, China; 126 VIB Center for Cancer Biology, 3001 Louvain, Belgium; 127 Laboratory for Translational Genetics, Department of Human Genetics, University of Leuven, 3000 Louvain, Belgium; 128 Epidemiology Program, University of Hawaii Cancer Center, Honolulu, HI 96813 USA; 129 Human Genetics Division, Genome Institute of Singapore, Singapore, 138672 Singapore; 130 Division of Epidemiology, Department of Medicine, Vanderbilt Epidemiology Center, Vanderbilt-Ingram Cancer Center, Vanderbilt University School of Medicine, Nashville, TN 37232 USA; 131 Division of Population Health, Health Services Research and Primary Care, School of Health Sciences, Faculty of Biology, Medicine and Health, The University of Manchester, Manchester, M13 9PL UK; 132 Biobank of Eastern Finland, Kuopio University Hospital, Kuopio, Finland; 133 Molecular Genetics of Breast Cancer, German Cancer Research Center (DKFZ), 69120 Heidelberg, Germany; 134 Department of Clinical Science and Education, Sšdersjukhuset, Karolinska Institutet, 118 83 Stockholm, Sweden; 135 Division of Cancer Epidemiology and Prevention, Aichi Cancer Center Research Institute, Nagoya, 464-8681 Japan; 136 Department of Medical Oncology, University Hospital of Heraklion, 711 10 Heraklion, Greece; 137 School of Population and Public Health, University of British Columbia, Vancouver, BC V6T 1Z4 Canada; 138 Cancer Control Research, BC Cancer, Vancouver, BC V5Z 1L3 Canada; 139 Department of Obstetrics and Gynecology, Helsinki University Hospital, University of Helsinki, 00290 Helsinki, Finland; 140 Department of Gynecology and Obstetrics, University Hospital Düsseldorf, Heinrich-Heine University Düsseldorf, 40225 Düsseldorf, Germany; 141 Epidemiology Branch, National Institute of Environmental Health Sciences, NIH, Research Triangle Park, NC 27709 USA; 142 Institute for Medical Biometry and Epidemiology, University Medical Center Hamburg-Eppendorf, 20246 Hamburg, Germany; 143 Clinical Genetics Research Lab, Department of Cancer Biology and Genetics, Memorial Sloan Kettering Cancer Center, New York, NY 10065 USA; 144 Clinical Genetics Service, Department of Medicine, Memorial Sloan Kettering Cancer Center, New York, NY 10065 USA; 145 Department of Epidemiology, Gillings School of Global Public Health and UNC Lineberger Comprehensive Cancer Center, University of North Carolina at Chapel Hill, Chapel Hill, NC USA; 146 Integrated Major in Innovative Medical Science, Seoul National University College of Medicine, Seoul, 03080 South Korea; 147 Department of Population Science, American Cancer Society, Atlanta, GA 30303 USA; 148 Department of Genetics, Lineberger Comprehensive Cancer Center, University of North Carolina at Chapel Hill, Chapel Hill, NC USA; 149 Department of Non-Communicable Disease Epidemiology, London School of Hygiene and Tropical Medicine, London, WC1E 7HT UK; 150 Research Centre for Genetic Engineering and Biotechnology “Georgi D. Efremov”, MASA, Skopje, 1000 Republic of North Macedonia; 151 Unit of Molecular Bases of Genetic Risk and Genetic Testing, Department of Research, Fondazione IRCCS Istituto Nazionale Dei Tumori (INT), 20133 Milan, Italy; 152 Department of Basic Sciences, Shaukat Khanum Memorial Cancer Hospital and Research Centre (SKMCH & RC), Lahore, 54000 Pakistan; 153 Medical Oncology Department, Hospital Universitario Puerta de Hierro, 28222 Madrid, Spain; 154 Department of Oncology, Mayo Clinic, Rochester, MN 55905 USA; 155 Department of Oncology, University Hospital of Larissa, 411 10 Larissa, Greece; 156 School of Cancer and Pharmaceutical Sciences, Comprehensive Cancer Centre, Guy’s Campus, King’s College London, London, SE1 9RT UK; 157 Division of Psychosocial Research and Epidemiology, The Netherlands Cancer Institute - Antoni Van Leeuwenhoek Hospital, Amsterdam, 1066 CX The Netherlands; 158 Center for Molecular Medicine Cologne (CMMC), Faculty of Medicine and University Hospital Cologne, University of Cologne, 50931 Cologne, Germany; 159 Department of Health Sciences Research, Mayo Clinic, Rochester, MN 55905 USA; 160 Department of Internal Medicine, Division of Medical Oncology, University of Kansas Medical Center, Westwood, KS 66205 USA; 161 Institute of Biomedical Sciences, Academia Sinica, Taipei, 115 Taiwan; 162 School of Public Health, China Medical University, Taichung, Taiwan; 163 Genomics Center, Centre Hospitalier Universitaire de Québec – Université Laval Research Center, Québec City, QC G1V 4G2 Canada; 164 Department of Population Health Sciences, Weill Cornell Medicine, New York, NY 10065 USA; 165 Epigenetic and Stem Cell Biology Laboratory, National Institute of Environmental Health Sciences, NIH, Research Triangle Park, NC 27709 USA; 166 Department of Surgery, Faculty of Medicine, University of Malaya, 50603 Kuala Lumpur, Malaysia; 167 Department of Cancer Biology and Genetics, The Ohio State University, Columbus, OH 43210 USA; 168 Department of Surgery, Leiden University Medical Center, Leiden, 2333 ZA The Netherlands; 169 Institute of Human Genetics, Pontificia Universidad Javeriana, 110231 Bogota, Colombia; 170 Center for Population Health Research, National Institute of Public Health, 62100 Cuernavaca, Morelos, Mexico; 171 Department of Quantitative Health Sciences, Division of Epidemiology, Mayo Clinic, Rochester, MN 55905 USA; 172 Biostatistics and Computational Biology Branch, National Institute of Environmental Health Sciences, NIH, Research Triangle Park, NC 27709 USA; 173 Laboratory of Cancer Genetics and Tumor Biology, Cancer and Translational Medicine Research Unit, Biocenter Oulu, University of Oulu, 90570 Oulu, Finland; 174 Laboratory of Cancer Genetics and Tumor Biology, Northern Finland Laboratory Centre Oulu, 90570 Oulu, Finland; 175 Department of Surgical Sciences, Uppsala University, 751 05 Uppsala, Sweden; 176 Department of Surgery, Tri-Service General Hospital, National Defense Medical Center, Taipei, 114 Taiwan; 177 Department of Medicine, Diller Family Comprehensive Cancer Center, Institute for Human Genetics, UCSF Helen, University of California San Francisco, San Francisco, CA 94115 USA; 178 Health Data Research UK, University College London, London, UK; 179 University College London Hospitals Biomedical Research Centre (UCLH BRC), London, UK; 180 The Alan Turing Institute, London, UK; 181 UCL Genetics Institute, University College London, London, UK

**Keywords:** Breast cancer susceptibility, Diverse ancestry, Rare variants, Gene regulation, Genome-wide association study

## Abstract

**Background:**

Low-frequency variants play an important role in breast cancer (BC) susceptibility. Gene-based methods can increase power by combining multiple variants in the same gene and help identify target genes.

**Methods:**

We evaluated the potential of gene-based aggregation in the Breast Cancer Association Consortium cohorts including 83,471 cases and 59,199 controls. Low-frequency variants were aggregated for individual genes’ coding and regulatory regions. Association results in European ancestry samples were compared to single-marker association results in the same cohort. Gene-based associations were also combined in meta-analysis across individuals with European, Asian, African, and Latin American and Hispanic ancestry.

**Results:**

In European ancestry samples, 14 genes were significantly associated (*q* < 0.05) with BC. Of those, two genes, *FMNL3* (*P* = 6.11 × 10^−6^) and *AC058822.1* (*P* = 1.47 × 10^−4^), represent new associations. High FMNL3 expression has previously been linked to poor prognosis in several other cancers. Meta-analysis of samples with diverse ancestry discovered further associations including established candidate genes *ESR1* and *CBLB*. Furthermore, literature review and database query found further support for a biologically plausible link with cancer for genes *CBLB, FMNL3, FGFR2*, *LSP1*, *MAP3K1*, and *SRGAP2C*.

**Conclusions:**

Using extended gene-based aggregation tests including coding and regulatory variation, we report identification of plausible target genes for previously identified single-marker associations with BC as well as the discovery of novel genes implicated in BC development. Including multi ancestral cohorts in this study enabled the identification of otherwise missed disease associations as *ESR1* (*P* = 1.31 × 10^−5^), demonstrating the importance of diversifying study cohorts.

**Supplementary Information:**

The online version contains supplementary material available at 10.1186/s13073-022-01152-5.

## Background

Breast cancer is the most commonly diagnosed cancer in women worldwide, making up 11.7% of new cancer diagnoses in 2020 [1]. Heritability estimates for breast cancer range from 13% [2] to 30% [3]. Breast cancer follows a predominantly complex genetic architecture, which in large parts remains unsolved to this day [4]. Identifying disease predisposing genes in breast cancer can help understand pathological pathways and discover new clinical biomarkers or drug targets. However, linking single-marker associations identified in genome-wide association studies (GWAS) to target genes is still ongoing [5], precluding better mechanistic disease understanding.

The analysis of data from diverse ancestral groups can uncover new insights about genetic risk factors due to ancestral differences in variant frequency and linkage disequilibrium patterns, especially in the context of low-frequency variants, as well as variation in environmental factors [6–8]. Thus, extending genetic studies to diverse populations and groups is a necessary advance to gain a comprehensive understanding of genetic architectures of complex diseases.

In this study, we extend the recently published gene aggregation method combining coding and regulatory variants [9] to large-scale whole-genome genotyping cohorts to uncover novel genes implicated in breast cancer development. We used data from the Breast Cancer Association Consortium (BCAC) which has been studied previously including GWAS [10, 11], candidate gene analysis [12], and polygenic risk score analysis [13, 14].

We employ the following strategies to empower the discovery of novel gene-disease associations using data from BCAC: (1) aggregation of all coding and regulatory variants linked to a single gene, (2) effective utilization of low-frequency variants, (3) exploiting genetic diversity between different ancestral groups, and (4) restricting multiple testing burden to one statistical test per gene (~ 18,500).

## Methods

### Samples and genotype data

**Fig. 1:**
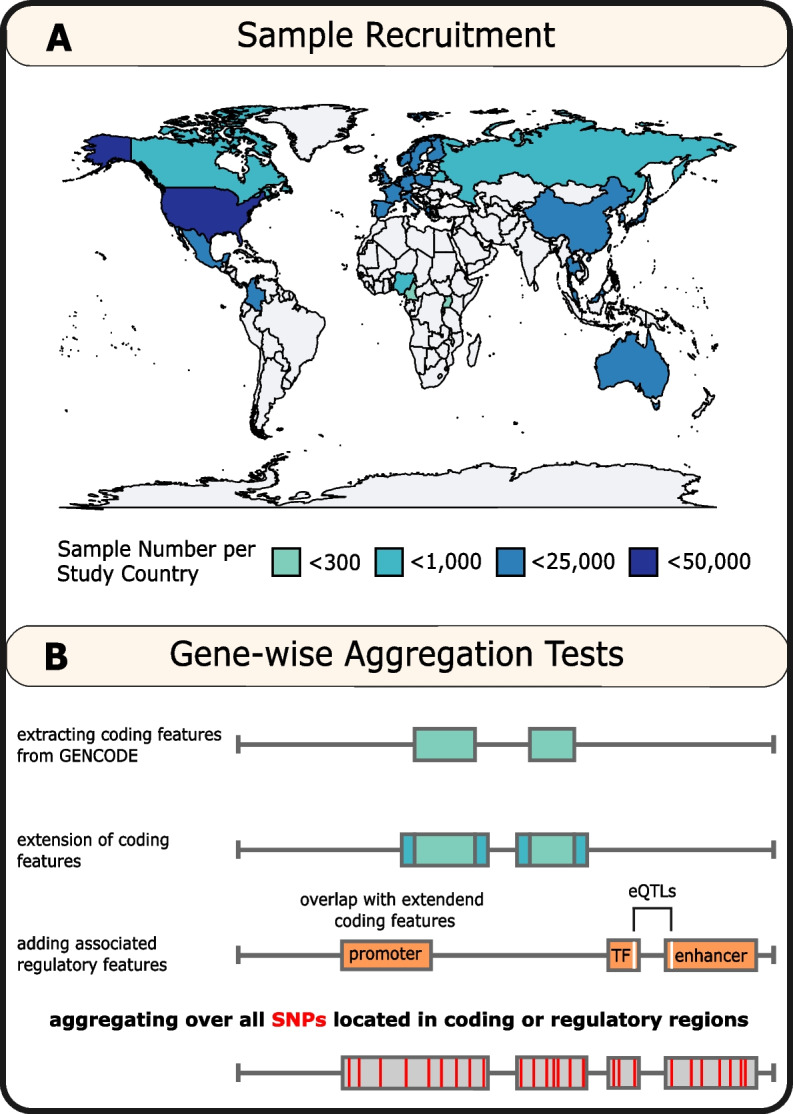
Study design. **A** Breast cancer patients and control individuals included in this study originate from 33 different study center countries, and comprise samples of African, Asian, European, or Latin American and Hispanic ancestry. **B** The mummy implemented extended SKAT-O analysis includes variants located in coding regions with an extended window and variants located in linked regulatory regions. Regulatory regions were identified based on overlap with genetic range of coding features or based on presence of gene-specific eQTLs in GTEx data in those regulatory regions

We used data on 142,670 individuals from BCAC. Detailed description of recruitment criteria, sample demographics, genotyping quality control, and imputation of additional markers have been reported previously [10, 15, 16]. In short, 83,471 breast cancer cases and 59,199 controls of diverse ancestry were recruited in 80 studies (see Fig. 1A, Additional file 1: Table S1). For each study, country of origin, and case and control numbers can be found in Additional file 1: Table S2. Samples were genotyped using the OncoArray (Illumina) [17], a custom SNP array enriched for cancer-associated genetic regions.

### Quality control of genotype data

Sample quality control based on genotype and imputation quality has been performed previously [10]. In short, samples were genotyped on the custom OncoArray. Genotyped markers failing any of the following quality criteria were excluded: (i) call rate above 98% in all consortia, (ii) MAF < 1%, (iii) no significant deviation from Hardy–Weinberg Equilibrium (controls: *P* < 10 − 7, cases: *P* < 10 − 12). Markers were imputed in a two-stage approach using shapeit2 and impute2 (V2) and the October 2014 (version 3) release of the 1000 Genomes dataset as reference panel [10]. The imputation was carried out for 5-Mb segments of the genome and for groups of 10,000 samples to reduce the computation burden. We included only low-frequency variants (minor allele frequency MAF < 0.05). Variants with imputation accuracy scores (generated with IMPUTE version 2) below 0.7 were excluded from analysis.

### Selection of genetic elements

Our previously developed analysis pipeline “mummy” [9] was used to identify coding and regulatory regions for individual genes and to prepare input data for robust rare variant SNPset association testing software MONSTER [18]. Aggregation tests were performed for genes defined in GENCODE v25 and with at least three but not more than 5000 low-frequency variants.

For each of these genes, we identified genetic elements that are likely to contain relevant functional or expression variation using the mummy wrapper. These include the exomes and untranslated regions (UTR) of the gene. We selected additional regulatory elements that have been shown to be enriched for complex trait associations [19, 20]: promoter, enhancer, and transcription-factor-binding units if they could be linked to the gene. These elements were identified from the Ensembl build 84 resource. The link of regulatory genetic elements to genes was either based on physical overlap with the coding region, e.g., when an element was located within an intron of the gene, or physical overlap with significantly associated eQTLs for the specific gene (see Fig. 1B). Thus, we included the three types of regulatory elements if there was evidence that they affect expression levels of the gene. This was based on eQTL data for all available cell types from GTEx version 6.

For each gene, all the low-frequency variants in these selected genetic elements were extracted and formatted to the MONSTER required input and weighted using Phred-scaled EigenPC pathogenicity scores [21]. EigenPC scores have been previously shown to offer the best balance between coding and noncoding variants for application in aggregation testing [9].

The original implementation of “mummy” was adapted to allow for the input of genotype data based on DNA microarrays instead of sequencing data in VCF format. The adapted “mummy” code is accessible on github here: https://github.com/stef-mueller/mummy_for_genotypes.

### Gene-based aggregation test

MONSTER (Minimum *P*‐value Optimized Nuisance parameter Score Test Extended to Relatives) was used to perform SNPset variant aggregation tests for the variants selected for each gene [18]. MONSTER generalizes the SKAT-O algorithm to allow for testing of related samples and sample cohorts with underlying population structure using a mixed effects model. SKAT-O is a unified test that combines a variance component and a burden test. The original MONSTER code was adapted to allow for the inclusion of larger sample numbers. The adapted MONSTER code is available on github here: https://github.com/stef-mueller/MONSTER.

Samples were processed in 15 study groupings due to the computational demand. Groups were formed based on study origin and genetic ancestry of samples while ensuring balanced case and control numbers. Additional file 1: Table S3 lists the number of analyzed genes for each cohort. Sample numbers per cohort can be found in Additional file 1: Table S4.

The mixed effects models testing for gene associations included relatedness in the form of a kinship matrix as a random effect. The kinship matrix was derived by, first, creating an LD pruned marker set using plink2 [22] (window size: 50 kb, step size: 5, *r*^2^ threshold: 0.5, minor allele frequency threshold: > 0.2), second, calculating a relationship matrix using gemma [23], third, calculating individuals’ inbreeding coefficients using plink2 –ibc command, and fourth, combining relationship matrix and inbreeding coefficients to the MONSTER required input format. Additionally, age and for some cohorts the recruitment study or study country were included in the model as fixed effects (Additional file 1: Table S5).

As is common for SNPset aggregation tests, MONSTER reports as output *P*-values but not effect sizes or effect directions for linear mixed model aggregation tests. To check for unaccounted population stratification effects, raw aggregation test results per cohort were plotted against the theoretical distribution of *P*-values using quantile–quantile (QQ) plots (see Additional file 1: Figure S1), and genetic inflation factors lambda and lambda1000 were calculated (see Additional file 1: Table S4). Lambda is dependent on sample size and will be increased for large samples. Lambda1000 has been established to be comparable across studies. It corrects for sample size.

Two of the 15 cohorts, one of European ancestry and the Latin American and hispanic group, were found to have increased genetic inflation factors with lambda1000 metrics of 1.32 and 1.14, respectively. Thus, raw aggregation test *P*-values for these two cohorts were corrected using the genomic control method.

### Meta-analysis of aggregation tests

Two meta-analyses were performed to combine raw aggregation association results from individual cohorts. First, to allow for comparison with the published GWAS [10] results based on the same sample set, all cohorts including samples of predominantly European ancestries (twelve cohorts, all named “eur*”) were combined in an all-European meta-analysis. Next, a second meta-analysis was performed including all cohorts.

The Stouffer [24, 25] method was used to perform the meta-analysis. It combines the *z*-statistic derived from *P*-values of the aggregate test for each cohort after weighting with the square root of the respective sample size. For cohorts with increased genetic inflation factor lambda1000, genetic control corrected *P*-values, rather than the raw *P*-values, were included in the meta-analysis. The R package metaP (version 1.3) was used to perform Stouffer meta-analysis. No evidence for increased inflation was observed for the meta-analysis results based on QQ plots and inflation estimates (Additional file 1: Figure S2).

Benjamini–Hochberg false discovery rate (FDR) method was used to correct the meta-analysis results for multiple testing. To ensure robust association signals, genes with missing results for the majority of cohorts were excluded from further analysis. Significant hits were defined as those with FDR-corrected *P*-values < 0.05.

### Follow-up on significantly associated genes

We evaluated whether any of the significant gene-based associations with breast cancer overlapped with significant single-marker associations arising from the European ancestry GWAS. The genome-wide association analysis for single markers in the European ancestry samples has been previously described [10]. The comparison was based on coding and regulatory regions of the gene-based hits with a flanking region of 100 kb. The flanking region of 100 kb was chosen to ensure inclusion of the majority of cis-eQTL elements which, based on GTEx data of 44 tissues, have a median distance of 28.9 or 50.1 kb from the transcription start site (TSS) of genes for primary and secondary cis-eQTLs, respectively [26]. Loci that included SNPs with *P*-values below 5 × 10^−8^ from the single-marker association analysis in the examined regions were classified as previously identified breast cancer association hits.

We carried out bioinformatic annotations for each significantly associated gene. Four open-source databases were queried for prior evidence of a causal role of the genes in breast cancer pathology specifically as well as any cancer pathology. First, the ClinVar database was used to identify any putative pathogenic, single-gene variants reported previously in the context of the phenotypes of interest. The ClinVar database was queried on the 1st of March 2021. Pathogenic, single-gene ClinVar variant entries with at least one star review status were classified as supportive evidence.

Second, the aggregated gene-disease database MalaCards [27] was used to identify any significant correlation of genes and phenotypes of interest based on 68 different data sources and utilizing NLP (Natural Language Processing) algorithms to include evidence from non-structured data sources like research publications. Supportive evidence of causal role of genes was defined as a MalaCards search relevance score over 1. The MalaCard database was queried on the 1st of March 2021.

Third, the expert-curated Genetics Home Reference data was queried for all genes of interest and examined for evidence of causal role in breast cancer or any cancer. The queried data version was published on the 28th of July 2020.

And fourth, investigating possible roles as driver genes in breast cancer and cancer pathogenicity, we queried the COSMIC Cancer Gene Census data (version 92) which classifies genes as either (1) TIER1: genes with strong evidence of causal role promoting cancer such as documented relevance in cancer and oncogenic mutations, (2) TIER2: genes with substantial indications to play a role in cancer etiology, and (3) untiered genes: genes with no substantial evidence of a causal role.

## Results

Gene-wise aggregation analysis was performed in 83,471 breast cancer patients and 59,199 matched controls. Of those 142,670 samples, 83.4% (*n* = 119,014) were of European ancestry, with 10.7% (*n* = 15,321) of samples being of Asian, 4.1% (*n* = 5784) of African, or 1.8% (*n* = 2551) Latin American and Hispanic ancestry, respectively. Samples were recruited to studies in 33 countries (see Fig. 1A).

### All-European meta-analysis finds 14 associated breast cancer genes

First, we combined gene-wise association results for European cohorts in an all-European meta-analysis. After multiple testing correction, we found 14 genes located in nine different regions to be significantly associated with breast cancer risk (Table 1). Overlap in coding and regulatory regions of genes can cause non-unique mapping of variants to multiple genes for the association aggregation test performed in MONSTER. Thus, four loci were identified containing more than one associated gene. Regional plots for all 14 genes can be found in Additional file 1: Figure S3.

**Table 1:** Meta-analysis hits in samples of European ancestry. Results for significant (*q* < 0.05) gene associations from the meta-analysis of 12 cohorts of European ancestry. Genes with overlapping coding and/or regulatory regions are summarized as a single locus defined as the intersection of all included genetic regions. Overlap with single-marker association results from Michailidou et al. [10] are also shown, with new associations identified for *FMNL3* and *AC058822.1*

Locus (hg38)	Stable gene ID	Gene	Unadjusted *P*-value	*q*-value	Michailidou (2017) GWAS association
chr5:56,815,574–56,971,675	ENSG00000095015	MAP3K1	4.61E − 22	8.28E − 18	Yes
	ENSG00000155545	MIER3	2.81E − 06	7.22E − 03	Yes
chr1:121,167,646–121,392,822	ENSG00000188610	FAM72B	1.32E − 15	1.19E − 11	Yes
	ENSG00000171943	SRGAP2C	1.01E − 14	6.07E − 11	Yes
chr11:1,852,970–1,938,706	ENSG00000130595	TNNT3	6.17E − 08	2.77E − 04	Yes
	ENSG00000130592	LSP1	1.31E − 07	4.70E − 04	Yes
chr10:121,478,334–121,598,458	ENSG00000066468	FGFR2	9.48E − 07	2.84E − 03	Yes
chr12:49,636,499–49,708,165	ENSG00000161791	FMNL3	6.11E − 06	1.37E − 02	No
chr19:43,766,533–43,901,385	ENSG00000104783	KCNN4	1.12E − 05	2.03E − 02	Yes
	ENSG00000159871	LYPD5	1.39E − 05	2.03E − 02	Yes
	ENSG00000176222	ZNF404	2.23E − 05	2.86E − 02	Yes
chr4:53,377,839–54,295,272	ENSG00000282278	AC058822.1	1.47E − 05	2.03E − 02	No
chr4:83,459,517–83,523,348	ENSG00000163322	ABRAXAS1	1.40E − 05	2.03E − 02	Yes
chr6:26,457,904–26,476,621	ENSG00000112763	BTN2A1	1.26E − 05	2.03E − 02	Yes

For twelve of the 14 associated genes, the region (gene plus a 100-kB flanking region) contained markers that were individually associated with breast cancer at genome-wide significance (*P*-value < 5 × 10^−8^).

### Two novel associations

**Fig. 2:**
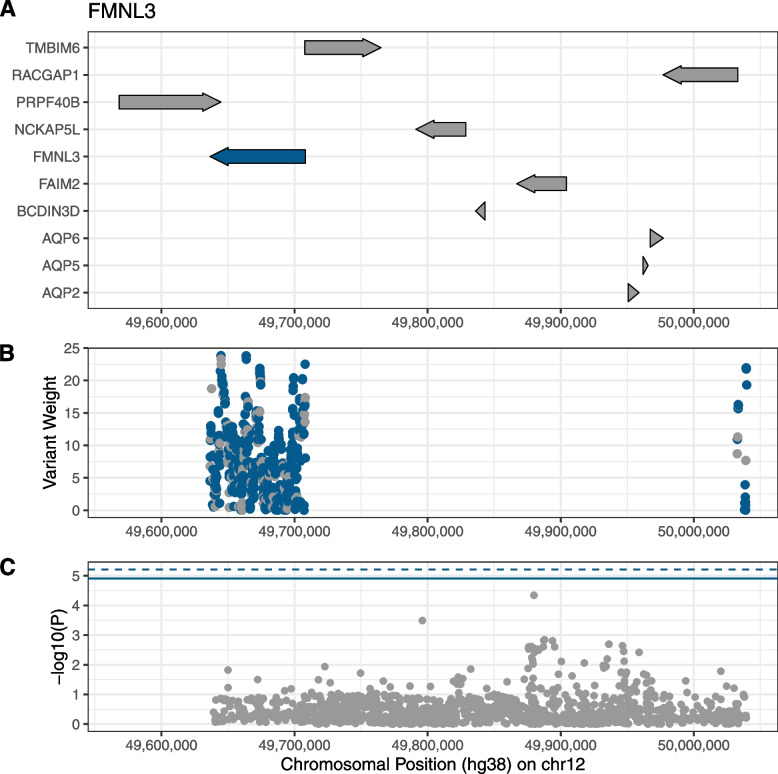
Regional Plot the *FMNL3* Gene on Chromosome 12. Regional plots for the breast cancer association of *FMNL3* at 12q13.12. **A** Depiction of coding regions of all coding genes (data retrieved from Ensembl biomart hg38) within the chromosomal region with *FMNL3* highlighted in blue. **B** Variants included in the aggregation test, plotted according to their chromosomal position and analysis weight. Highlighted in blue are variants exclusively present in the analysis of samples of diverse ancestry. **C** Single-marker association results based on the same samples [10], with blue solid line denoting *P*-value for meta-analysis of all cohorts for gene of interest (*P* = 1.24 × 10^−5^) in this study and blue dashed line denoting unadjusted *P*-value for all-European meta-analysis (*P* = 6.11 × 10^−6^)

The gene-wise aggregation of low-frequency variants based on coding and regulatory features was able to extend findings of a standard GWAS analysis. The analysis identified two novel gene associations that do not overlap previously reported single-marker-based loci (Fig. 2). The *FMNL3* (Formin-Like 3) gene at 12q13.12 was associated with breast cancer risk with a *q*-value of 0.013. It encodes the Formin-like protein 3, a cytoskeletal regulator, whose overexpression is associated with cancer cell migration, invasion, metastasis, and poor prognosis in multiple cancer types, such as colorectal carcinoma [28], nasopharyngeal carcinoma [29], and tongue squamous cell carcinoma [30].

The second novel association was found at 4q12 for *AC058822.1* (*q*-value = 0.020), also named *RP11-231C18.3*. This lncRNA gene is a scarcely characterized genetic element spanning almost 1 MB.

### Gene-based aggregation can help identify the causal genes

To assess whether the gene-based approach can help highlight biologically plausible gene candidates, we assessed whether other evidence, such as genetic epidemiological studies or cell models, supports a role for the significantly associated genes in cancer. We queried different public databases for links to breast cancer and other cancer types for the 14 genes found to be associated with breast cancer in the all-European meta-analysis.

Two genes, *MAP3K1* and *FGFR2*, in addition to being previously identified in breast cancer-associated genetic region in GWAS (see Table 2), are both classified as TIER1 cancer-driving genes in COSMIC Cancer Gene Census. Thus, there is strong evidence that somatic mutations in both genes have a functional involvement in cancer etiology.

**Table 2:** Support for a Role in Cancer for the 14 Associated Genes. Prior supportive evidence for genes associated with breast cancer in the aggregation test was based on presence of pathogenic cancer mutations in those, based on ClinVar and curated genetic reference database Genetics Home Reference and aggregation database Malacards. In addition, hit genes were queried in the COSMIC Cancer Gene Census database

		**Causal evidence in breast cancer**			**Causal evidence in any cancer**			
**Locus (hg38)**	**Gene**	**ClinVar**	**Genetics Home** **Reference**	**Malacards** **Score > 1**	**ClinVar**	**Genetics Home** **Reference**	**Malacards** **Score > 1**	**Cancer Gene Census** **[TIER1;TIER2;NO]**
chr1:121,167,646–121,392,822	FAM72B	NO	NO	NO	NO	NO	NO	NO
	SRGAP2C	NO	NO	NO	NO	NO	YES	NO
chr4:53,377,839–54,295,272	AC058822.1	NA	NA	NA	NA	NA	NA	NA
chr4:83,459,517–83,523,348	ABRAXAS1^a^	NO	NO	NO	NO	NO	NO	NO
chr5:56,815,574–56,971,675	MAP3K1	NO	YES	YES	YES	NO	YES	TIER1
	MIER3	NO	NO	NO	NO	NO	NO	NO
chr6:26,457,904–26,476,621	BTN2A1	NO	NO	NO	NO	NO	NO	NO
chr10:121,478,334–121,598,458	FGFR2	NO	YES	YES	YES	YES	YES	TIER1
chr11:1,852,970–1,938,706	TNNT3	NO	NO	NO	NO	NO	NO	NO
	LSP1	NO	NO	NO	NO	NO	YES	NO
chr12:49,636,499–49,708,165	FMNL3	NO	NO	NO	NO	NO	YES	NO
chr19:43,766,533–43,901,385	KCNN4	NO	NO	NO	NO	NO	NO	NO
	LYPD5	NO	NO	NO	NO	NO	NO	NO
	ZNF404	NO	NO	NO	NO	NO	NO	NO

Gene AC058822.1 was not present in the queried databases

^a^*ABRAXAS1* was additionally queried using the alias *FAM175A*

To search for previous causal evidence of germline mutations in associated genes, we queried ClinVar, Genetic Home Reference, and MalaCards databases—the last two being an expert-curated gene-disease database and an aggregation database of 68 data sources, respectively. Five genes were implicated in the development of other cancer types: *SRGAP2C*, *MAP3K1*, *FGFR2*, *LSP1*, and *FMNL3*.

In addition, the gene *ABRAXAS1* codes for a subunit of the BRCA1-A complex [31]. This protein complex plays an important role in DNA damage repair and mutations in the *BRCA1* gene predispose to increased risks of cancer [32].

In summary, we found support for aggregated gene associations coinciding with prior causal evidence in breast cancer for two of the nine associated genes and in any cancer for five of them. Among the four associated genes without or very limited prior evidence in cancer pathophysiology is the single-gene locus spanning gene *ABRAXAS1*—a promising candidate gene for further follow-up owing to its close interactions with protein BRCA1 and its role in DNA damage repair [33].

### Including ancestrally diverse samples finds additional gene associations

We furthermore tested gene-based associations in the African (*n* = 5784), Asian (*n* = 15,321), and Latin American and Hispanic (*n* = 2551) ancestry cohorts. There were no significant associations after FDR multiple testing correction. We considered suggestive associations with unadjusted, or in case of the Latin American and Hispanic cohort genetic control corrected, *P*-values below 1 × 10^−4^. While no suggestive associations were found in the Latin American and Hispanic cohort, four and five gene associations could be identified in the African and Asian cohort, respectively (Additional file 1: Table S7 and Table S8). This included a suggestive association of gene *CBLB* (unadjusted *P*-value: 2.11 × 10^−5^, Additional file 1: Figure S5) in the African cohort. The E3 Ubiquitin Ligase Cbl-b, coded by oncogene *CBLB*, has been reported to affect cancer development and progression [34] and has been proposed as a clinical biomarker in breast cancer [35]. No variants located in the coding region of *CBLB* (plus 100 kb flanking region) were found to be associated in the 2017 large-scale GWAS [10]. None of the variants at this locus have been previously linked to any breast cancer phenotype based on the GWAS Catalog. Thus, the inclusion of diverse ancestry samples shows promise for the identification of new suggestive associations for a plausible candidate gene.

In a second meta-analysis, all 15 sample cohorts, including European ancestry cohorts and cohorts of Asian, African, or Latin American and Hispanic ancestry, were combined (Additional file 1: Table S6). This analysis identified an additional association of gene *ESR1* (FDR adjusted *P*-value in all cohort meta-analysis: 0.0269; Additional file 1: figure S4). The gene *ESR1* codes for the estrogen receptor alpha protein and genetic variations in this gene have been reported to be associated with breast cancer [10, 36] and are well described in breast cancer etiology [37] impacting cancer progression [38], treatment success [39], and long term disease outcomes [40].

## Discussion

We report the results of a gene-based association analysis in the BCAC resource. Adopting a recently proposed aggregation method that combines variants in coding and regulatory regions, we were able to replicate and extend previously reported findings. This aggregation method helps identify target genes of previously reported single-marker associations and uncovers additional associations that were missed by other methods.

We found 14 genes located in nine loci to be significantly associated with breast cancer risk in samples of European ancestry. Variants near seven of these loci have previously been implicated in breast cancer development based on the 2017 GWAS by Michailidou et al. [10] and we were able to link those single-marker associations to putative target genes. We found independent evidence for a role in breast cancer development for five of the genes. Two of them, *MAP3K1* and *FGFR2*, are long-established risk genes for breast cancer mediated by both germline and somatic mutations [41, 42]. MAP kinase MEKK1, coded by *MAP3K1*, has been reported to promote cancer cell migration by contributing to an accommodating breast tumor microenvironment [43, 44], while *FGFR2* has been identified as a viable drug target in breast cancer [45]. Additionally, the genes *SRGAP2C*, *LSP1*, and *FMNL3* have been implicated in the etiology of other types of cancer. Although there is currently no functional evidence to substantiate the role of these three genes in breast cancer, sharing of genetic risk factors between different cancers is prevalent [46]. Jiang et al. report a genetic correlation of 0.24, 0.18, and 0.15 for breast cancer with ovarian, lung, and colorectal cancer, respectively [2].

As a further plausible target gene, we have identified *ABRAXAS1*, which codes for a subunit of the BRCA1 DNA repair protein complex. Differential allelic expression in the genomic region 4q21, in which gene ABRAXAS1 is located, has been previously reported to be associated with breast cancer susceptibility [47]. Interestingly, a recent study using burden testing for rare, protein-truncating or pathogenic variants in *ABRAXAS1* based on sequencing data from 60,000 patients and 53,000 controls from the BCAC cohort did not find a significant disease association, with the odds ratio reported as 0.98 (0.50–1.94) [12]. In contrast, our approach focusing on low-frequency coding and regulatory variants identified a significant association of this gene with breast cancer risk. This suggests that our method enables gene discoveries that are missed by other approaches because the local genetic architecture of genes affecting breast cancer susceptibility varies between ancestry groups.

Beyond the identification of putative target genes in loci that have been previously found to harbor disease-associated variants, we report here two new disease associations for genes *FMNL3* and *AC058822.1*. *FMNL3* is a member of the diaphanous-related formin family, which represents a family of highly conserved cytoskeletal regulatory proteins [48]. *FMNL3* expression is reported to promote migration and invasion of cancer cells and predicts clinical outcome in different solid cancers such as colorectal carcinoma [28, 49], squamous cell carcinoma of the tongue [30], and melanoma [50]. No markers in the proximity of this gene were found to be associated with breast cancer in the 2017 GWAS in the same dataset.

Features of the method that may facilitate discoveries beyond those identified by other approaches include (i) a reduction of multiple testing burden, (ii) boosting signals by aggregating over all genetic regions affecting individual genes expression and function, (iii) inclusion of low-frequency variants often underpowered in other studies, and (iv) ability to synthesize evidence for genetic risk factors in different ancestries regardless of differences in non-disease-associated variational background.

The inclusion of samples of non-European ancestry in genetic studies can advance our understanding of genetic disease landscapes [8]. However, differences between populations in terms of allele frequencies and linkage disequilibrium can lead to heterogeneity and false positive associations in single-marker association analyses. Additionally, different causal variants may be present in different ancestral groups [51] which can be driven by ancestry differences in allele frequencies. Aggregation methods offer a solution because they can accommodate multiple causal variants at a locus. A meta-analysis including all cohorts in this study was able to identify an additional association for *ESR1*, which was not detected in a European ancestry only analysis. Ancestry-related differences in disease-associated variants and minor allele frequencies in the ESR1 locus (6q25 region) have been previously reported [52, 53]. This *ESR1* gene is coding for the estrogen receptor alpha monomer, an established risk factor and promising clinical biomarker in breast cancer pathophysiology [37, 54, 55].

The comparably small sample size of cohorts of non-European ancestry is a limitation of our study. Although no gene reached FDR-corrected significance in these analyses, nine genes were associated at suggestive thresholds, including biologically plausible candidate gene *CBLB*. This gene codes for the E3 Ubiquitin Ligase Cbl-b, which is a confirmed protagonist in cancer development and progression [56, 57]. There is recently mounting evidence that CBLB expression may be useful as a prognostic factor in breast cancer [35, 58, 59].

We note the following limitations for the adopted method in this study. First, no effect sizes or effect directions are derived. Second, it is not clear how statistical power for identification of associations is affected by gene length, mutational constrictions, number of transcripts, and amount of prior evidence for regulatory elements. Future analyses could deliver insights in this regard. Third, we were not always able to narrow down associations to a single target gene in loci due to overlapping genetic features. This limitation is affected by the LD structure in a specific region and the amount of prior information available in form of eQTL data and regions of overlapping transcripts. Fourth, although we are able to find plausible target genes applying this method to samples of diverse ancestry, there is potential for further optimisation. Regulatory features for genes have been identified using GTEx data, which predominantly is derived from European ancestry samples. Additionally, variants are weighted using Phred-scaled EigenPC pathogenicity scores [21]. These scores are derived using unsupervised learning on a labeled training dataset predominantly based on samples of European descent. Fifth, the current implementation of the method is computationally demanding but nonetheless able to analyze large sample sets (here over 140,000 samples). Sixth, our analysis did not consider different transcripts of genes so our findings are limited to the assigned major transcript. And lastly, the optimal aggregate methods depend on the genetic architecture at a given locus. We used SKAT-O a unified test to capture a range of different architectures. However, the choice of method may impact on the results.

## Conclusions

Our findings show that usage of extended gene aggregation methods covering coding and regulatory regions in addition to standard single-marker tests (i.e., GWAS) have the potential to discover novel associations in available datasets. This study helps uncover the role of low-frequency genetic variation in breast cancer susceptibility and empowers gene discovery in ancestrally diverse cohorts.

## Supplementary Information

**Additional file 1:Table S1.** Cohort Descriptives. **Table S2.** Included BCAC studies. **Table S3.** Number of genes analysed per cohort. **Table S4.** Genomic inflation in burden analysis. **Table S5.** Model covariates per cohort. **Figure S1.** QQ-plots of burden analysis. **Figure S2.** QQ-plots for meta-analysis. **Figure S3.** Regional plots for significantly associated genes. FAM72B, SRGAP2C, AC058822.1, ABRAXAS1, MAP3K1, MIER3, BTN2A1, FGFR2, TNNT3, LSP1, LYPD5, KCNN4, ZNF404. **Table S6.** All Cohort Meta-Analysis. **Table S7.** Suggestive Associations in Diverse Ancestries. **Table S8.** Prior Evidence for Suggestive Associations in Diverse Ancestries. **Figure S4.** Regional plot for gene ESR1. **Figure S5.** Regional plot for gene CBLB. **Table S9.** Ethics committees that provided approval for the contributing studies.

## Data Availability

Gene aggregation results for all genes and all analyses, as well as code used in the analysis for this manuscript, are made available in the following github repository: https://github.com/stef-mueller/BCAC_genotype_aggregation_analysis [60]. Code for running mummy on genotypes available in public github repository here: https://github.com/stef-mueller/mummy_for_genotypes. An implementation of MONSTER, adapted for analyzing large-scale genotype data, is accessible on github: https://github.com/stef-mueller/MONSTER. Annotation sources used in this project are (1) ClinVar, https://www.ncbi.nlm.nih.gov/clinvar/; (2) MalaCards, https://www.malacards.org/; (3) Genetics Home Reference, https://medlineplus.gov/genetics/; (4) COSMIC Cancer Gene Census data, https://cancer.sanger.ac.uk/census. Summary statistics of GWAS data for breast cancer are available through the BCAC website: http://bcac.ccge.medschl.cam.ac.uk. The individual level datasets analyzed during the current study are not publicly available due to protection of participant privacy and confidentiality, and ownership of the contributing institutions, but may be made available in an anonymized form via the corresponding author on reasonable request and after approval of the involved institutions. To receive access to the data, a concept form must be submitted, which will then be reviewed by the BCAC Data Access Coordination Committee (DACC); see http://bcac.ccge.medschl.cam.ac.uk/bcacdata/. This work was carried out under the approved BCAC concept form #595.

## References

[1] Sung H, Ferlay J, Siegel RL, Laversanne M, Soerjomataram I, Jemal A (2021). Global Cancer Statistics 2020: GLOBOCAN estimates of incidence and mortality worldwide for 36 cancers in 185 countries. CA Cancer J Clin.

[2] Jiang X, Finucane HK, Schumacher FR, Schmit SL, Tyrer JP, Han Y (2019). Shared heritability and functional enrichment across six solid cancers. Nat Commun..

[3] Möller S, Mucci LA, Harris JR, Scheike T, Holst K, Halekoh U (2016). The heritability of breast cancer among women in the nordic twin study of cancer. Cancer Epidemiol Biomarkers Prev.

[4] Skol AD, Sasaki MM, Onel K (2016). The genetics of breast cancer risk in the post-genome era: thoughts on study design to move past BRCA and towards clinical relevance. Breast Cancer Res.

[5] Fachal L, Aschard H, Beesley J, Barnes DR, Allen J, Kar S (2020). Fine-mapping of 150 breast cancer risk regions identifies 191 likely target genes. Nat Genet.

[6] Kuchenbaecker K, Telkar N, Reiker T, Walters RG, Lin K, Eriksson A (2019). The transferability of lipid loci across African, Asian and European cohorts. Nat Commun.

[7] Wojcik GL, Graff M, Nishimura KK, Tao R, Haessler J, Gignoux CR (2019). Genetic analyses of diverse populations improves discovery for complex traits. Nature.

[8] Peterson RE, Kuchenbaecker K, Walters RK, Chen CY, Popejoy AB, Periyasamy S (2019). Genome-wide association studies in ancestrally diverse populations: opportunities, methods, pitfalls, and recommendations. Cell.

[9] Gilly A, Suveges D, Kuchenbaecker K, Pollard M, Southam L, Hatzikotoulas K (2018). Cohort-wide deep whole genome sequencing and the allelic architecture of complex traits. Nature Commun.

[10] Michailidou K, Lindström S, Dennis J, Beesley J, Hui S, Kar S (2017). Association analysis identifies 65 new breast cancer risk loci. Nature.

[11] Zhang H, Ahearn TU, Lecarpentier J, Barnes D, Beesley J, Qi G (2020). Genome-wide association study identifies 32 novel breast cancer susceptibility loci from overall and subtype-specific analyses. Nat Genet.

[12] Dorling L, Carvalho S, Allen J, González-Neira A, Luccarini C, Breast Cancer Association Consortium (2021). Breast cancer risk genes - association analysis in more than 113,000 women. N Engl J Med.

[13] Kramer I, Hooning MJ, Mavaddat N, Hauptmann M, Keeman R, Steyerberg EW (2020). Breast Cancer Polygenic Risk Score and Contralateral Breast Cancer Risk. Am J Hum Genet.

[14] Mavaddat N, Michailidou K, Dennis J, Lush M, Fachal L, Lee A (2019). Polygenic risk scores for prediction of breast cancer and breast cancer subtypes. Am J Hum Genet.

[15] Michailidou K, Hall P, Gonzalez-Neira A, Ghoussaini M, Dennis J, Milne RL (2013). Large-scale genotyping identifies 41 new loci associated with breast cancer risk. Nat Genet.

[16] Michailidou K, Beesley J, Lindstrom S, Canisius S, Dennis J, Lush MJ (2015). Genome-wide association analysis of more than 120,000 individuals identifies 15 new susceptibility loci for breast cancer. Nat Genet.

[17] Amos CI, Dennis J, Wang Z, Byun J, Schumacher FR, Gayther SA (2017). The OncoArray Consortium: a network for understanding the genetic architecture of common cancers. Cancer Epidemiol Biomarkers Prev.

[18] Jiang D, McPeek MS (2014). Robust rare variant association testing for quantitative traits in samples with related individuals. Genet Epidemiol.

[19] Finucane HK, Bulik-Sullivan B, Gusev A, Trynka G, Reshef Y, Loh PR (2015). Partitioning heritability by functional annotation using genome-wide association summary statistics. Nat Genet.

[20] Nasser J, Bergman DT, Fulco CP, Guckelberger P, Doughty BR, Patwardhan TA (2021). Genome-wide enhancer maps link risk variants to disease genes. Nature.

[21] Ionita-Laza I, McCallum K, Xu B, Buxbaum JD (2016). A spectral approach integrating functional genomic annotations for coding and noncoding variants. Nat Genet.

[22] Chang CC, Chow CC, Tellier LC, Vattikuti S, Purcell SM, Lee JJ (2015). Second-generation PLINK: rising to the challenge of larger and richer datasets. Gigascience.

[23] Zhou X, Stephens M (2012). Genome-wide efficient mixed-model analysis for association studies. Nat Genet.

[24] Stouffer SA, Suchman EA, Devinney LC, Star SA, Williams RM Jr. The American soldier: Adjustment during army life. (Studies in social psychology in World War II). Princeton Univ. Press; 1949.

[25] Zaykin DV (2011). Optimally weighted Z-test is a powerful method for combining probabilities in meta-analysis. J Evol Biol.

[26] GTEx Consortium (2017). Genetic effects on gene expression across human tissues. Nature.

[27] Rappaport N, Twik M, Plaschkes I, Nudel R, Stein TI, Levitt J, et al. MalaCards: an amalgamated human disease compendium with diverse clinical and genetic annotation and structured search, Nucleic Acids Research. 2017;45(D1):D877–D887. 10.1093/nar/gkw1012. 10.1093/nar/gkw1012 PMC5210521 27899610

[28] Zeng YF, Xiao YS, Lu MZ, Luo XJ, Hu GZ, Deng KY (2015). Increased expression of formin-like 3 contributes to metastasis and poor prognosis in colorectal carcinoma. Exp Mol Pathol.

[29] Wu Y, Shen Z, Wang K, Ha Y, Lei H, Jia Y (2017). High FMNL3 expression promotes nasopharyngeal carcinoma cell metastasis: role in TGF-β1-induced epithelia-to-mesenchymal transition. Sci Rep.

[30] Liu J, Chen S, Chen Y, Geng N, Feng C (2019). High expression of FMNL3 associates with cancer cell migration, invasion, and unfavorable prognosis in tongue squamous cell carcinoma. J Oral Pathol Med.

[31] Wang B, Matsuoka S, Ballif BA, Zhang D, Smogorzewska A, Gygi SP, Elledge SJ. Abraxas and RAP80 form a BRCA1 protein complex required for the DNA damage response. Science. 2007;316(5828):1194–8. 10.1126/science.1139476. 10.1126/science.1139476 PMC3573690 17525340

[32] Kuchenbaecker KB, Hopper JL, Barnes DR, Phillips KA, Mooij TM, Roos-Blom MJ (2017). Risks of breast, ovarian, and contralateral breast cancer for BRCA1 and BRCA2 mutation carriers. JAMA.

[33] Solyom S, Aressy B, Pylkäs K, Patterson-Fortin J, Hartikainen JM, Kallioniemi A (2012). Breast cancer-associated Abraxas mutation disrupts nuclear localization and DNA damage response functions. Sci Transl Med.

[34] Liyasova MS, Ma K, Lipkowitz S (2015). Molecular pathways: Cbl proteins in tumorigenesis and antitumor immunity-opportunities for cancer treatment. Clin Cancer Res.

[35] Liu X, Teng Y, Wu X, Li Z, Bao B, Liu Y (2020). The E3 ubiquitin ligase Cbl-b predicts favorable prognosis in breast cancer. Front Oncol.

[36] Milne RL, Kuchenbaecker KB, Michailidou K, Beesley J, Kar S, Lindström S (2017). Identification of ten variants associated with risk of estrogen-receptor-negative breast cancer. Nat Genet.

[37] Dustin D, Gu G, Fuqua SAW (2019). ESR1 mutations in breast cancer. Cancer.

[38] Lei JT, Shao J, Zhang J, Iglesia M, Chan DW, Cao J (2018). Functional annotation of ESR1 gene fusions in estrogen receptor-positive breast cancer. Cell Rep.

[39] Santo ID, De Santo I, McCartney A, Migliaccio I, Di Leo A, Malorni L (2019). The emerging role of ESR1 mutations in luminal breast cancer as a prognostic and predictive biomarker of response to endocrine therapy. Cancers.

[40] Zundelevich A, Dadiani M, Kahana-Edwin S, Itay A, Sella T, Gadot M (2020). ESR1 mutations are frequent in newly diagnosed metastatic and loco-regional recurrence of endocrine-treated breast cancer and carry worse prognosis. Breast Cancer Res.

[41] Stephens PJ, Tarpey PS, Davies H, Van Loo P, Greenman C, Wedge DC (2012). The landscape of cancer genes and mutational processes in breast cancer. Nature.

[42] Easton DF, Pooley KA, Dunning AM, Pharoah PDP, Thompson D, Ballinger DG (2007). Genome-wide association study identifies novel breast cancer susceptibility loci. Nature.

[43] Gentile S, Eskandari N, Rieger MA, Cuevas BD (2021). MEKK1 regulates chemokine expression in mammary fibroblasts: implications for the breast tumor microenvironment. Front Oncol.

[44] Cuevas BD, Winter-Vann AM, Johnson NL, Johnson GL (2006). MEKK1 controls matrix degradation and tumor cell dissemination during metastasis of polyoma middle-T driven mammary cancer. Oncogene.

[45] Chae YK, Hong F, Vaklavas C, Cheng HH, Hammerman P, Mitchell EP (2020). Phase II study of AZD4547 in patients with tumors harboring aberrations in the fgfr pathway: results from the NCI-MATCH Trial (EAY131) Subprotocol W. J Clin Oncol.

[46] Rashkin SR, Graff RE, Kachuri L, Thai KK, Alexeeff SE, Blatchins MA (2020). Pan-cancer study detects genetic risk variants and shared genetic basis in two large cohorts. Nat Commun.

[47] Hamdi Y, Soucy P, Adoue V, Michailidou K, Canisius S, Lemaçon A (2016). Association of breast cancer risk with genetic variants showing differential allelic expression: Identification of a novel breast cancer susceptibility locus at 4q21. Oncotarget.

[48] Katoh M, Katoh M (2003). Identification and characterization of human FMNL1, FMNL2 and FMNL3 genes in silico. Int J Oncol.

[49] Zeng YF, Xiao YS, Liu Y, Luo XJ, Wen LD, Liu Q (2018). Formin-like 3 regulates RhoC/FAK pathway and actin assembly to promote cell invasion in colorectal carcinoma. World J Gastroenterol.

[50] Gardberg M, Heuser VD, Koskivuo I, Koivisto M, Carpén O (2016). FMNL2/FMNL3 formins are linked with oncogenic pathways and predict melanoma outcome. Hip Int.

[51] Gelernter J, Sun N, Polimanti R, Pietrzak RH, Levey DF, Lu Q (2019). Genome-wide association study of maximum habitual alcohol intake in >140,000 U.S. European and African American Veterans Yields Novel Risk Loci. Biol Psychiatry.

[52] Fejerman L, Ahmadiyeh N, Hu D, Huntsman S, Beckman KB, Caswell JL (2014). Genome-wide association study of breast cancer in Latinas identifies novel protective variants on 6q25. Nat Commun.

[53] Hoffman J, Fejerman L, Hu D, Huntsman S, Li M, John EM (2019). Identification of novel common breast cancer risk variants at the 6q25 locus among Latinas. Breast Cancer Res.

[54] Dunning AM, Michailidou K, Kuchenbaecker KB, Thompson D, French JD, Beesley J (2016). Breast cancer risk variants at 6q25 display different phenotype associations and regulate ESR1, RMND1 and CCDC170. Nat Genet.

[55] Carausu M, Bidard FC, Callens C, Melaabi S, Jeannot E, Pierga JY (2019). ESR1 mutations: a new biomarker in breast cancer. Expert Rev Mol Diagn.

[56] Paolino M, Choidas A, Wallner S, Pranjic B, Uribesalgo I, Loeser S (2014). The E3 ligase Cbl-b and TAM receptors regulate cancer metastasis via natural killer cells. Nature.

[57] Liyasova MS, Ma K, Lipkowitz S (2015). Molecular pathways: cbl proteins in tumorigenesis and antitumor immunity-opportunities for cancer treatment. Clin Cancer Res.

[58] Xu L, Zhang Y, Qu X, Che X, Guo T, Cai Y (2017). E3 ubiquitin ligase Cbl-b prevents tumor metastasis by maintaining the epithelial phenotype in multiple drug-resistant gastric and breast cancer cells. Neoplasia.

[59] CHe X, Zhang Y, Qu X, Guo T, Ma Y, Li C (2017). The E3 ubiquitin ligase Cbl-b inhibits tumor growth in multidrug-resistant gastric and breast cancer cells. Neoplasma.

[60] Mueller HS (2022). Gene-aggregation results for all genes and all analyses generated in context of this project, github.

