# Supplementary material for: Aggregation tests identify new gene associations with breast cancer in populations with diverse ancestry
Source: Genome Med. 2023 Jan 26;15:7. doi: 10.1186/s13073-022-01152-5 (PMC9878779; doi:10.1186/s13073-022-01152-5)
Supplement: Supplementary file 1 — Additional file 1:Table S1. Cohort Descriptives. Table S2. Included BCAC studies. Table S3. Number of genes analysed per cohort. Table S4. Genomic inflation in burden analysis. Table S5. Model covariates per cohort. Figure S1. QQ-plots of burden analysis. Figure S2. QQ-plots for meta-analysis. Figure S3. Regional plots for significantly associated genes. FAM72B, SRGAP2C, AC058822.1, ABRAXAS1, MAP3K1, MIER3, BTN2A1, FGFR2, TNNT3, LSP1, LYPD5, KCNN4, ZNF404. Table S6. All Cohort Meta-Analysis. Table S7. Suggestive Associations in Diverse Ancestries. Table S8. Prior Evidence for Suggestive Associations in Diverse Ancestries. Figure S4. Regional plot for gene ESR1. Figure S5. Regional plot for gene CBLB. Table S9. Ethics committees that provided approval for the contributing studies. [file 13073_2022_1152_MOESM1_ESM.docx]

Additional file 1

##

[Supplemental Displays](#_1fob9te) **2**

[Table S1: Cohort Descriptives](#_3znysh7) 2

[Table S2: Included BCAC studies](#_tyjcwt) 3

[Table S3: Number of genes analysed per cohort](#_1t3h5sf) 6

[Table S4: Genomic inflation in burden analysis](#_4d34og8) 7

[Table S5: Model covariates per cohort](#_2s8eyo1) 8

[Figure S1: QQ-plots of burden analysis](#_3rdcrjn) 9

[Figure S2: QQ-plots for meta-analysis](#_26in1rg) 10

[Figure S3: Regional plots for significantly associated genes](#_lnxbz9) 11

[FAM72B](#_35nkun2) 11

[SRGAP2C](#_1ksv4uv) 12

[AC058822.1](#_44sinio) 13

[ABRAXAS1](#_z337ya) 14

[MAP3K1](#_3j2qqm3) 15

[MIER3](#_4i7ojhp) 16

[BTN2A1](#_2xcytpi) 17

[FGFR2](#_1ci93xb) 17

[TNNT3](#_3whwml4) 19

[LSP1](#_2bn6wsx) 20

[LYPD5](#_qsh70q) 21

[KCNN4](#_3as4poj) 22

[ZNF404](#_1pxezwc) 23

[Table S6: All Cohort Meta-Analysis](#_49x2ik5) 24

[Table S7: Suggestive Associations in Diverse Ancestries](#_2p2csry) 25

[Table S8: Prior Evidence for Suggestive Associations in Diverse Ancestries](#_3o7alnk) 26

[Figure S4: Regional plot for gene ESR1](#_ihv636) 27

[Figure S5: Regional plot for gene CBLB](#_32hioqz) 28

[Table S9: Ethics committees that provided approval for the contributing studies](#_8ndh4hxezdv2) 30

[Web Resources](#_yf70jtopyjoz) 33

[Supplemental References](#_hai7s6nzo0gv) 34

# Supplemental Displays

## Table S1: Cohort Descriptives

Description of samples included in analysis. Sample ancestry was determined based on genotyped data previously described in [(1)](https://paperpile.com/c/EH80kH/yI6Dc)

| **Phenotype** | **Control** | **Case** |
| --- | --- | --- |
| Sample Number | 59,199 | 83,471 |
| Female samples (%) | 100% | 100% |
| Age (median (IQR)) | 55.6 (16.35) | 55 (16.49) |
| Sample Number  African Ancestry (%) | 2,068 (3.5%) | 3,716 (4.5%) |
| Sample Number  Asian Ancestry (%) | 6,881 (11.6%) | 8,440 (10.1%) |
| Sample Number  European Ancestry (%) | 49,034 (82.9%) | 69,980 (83.8%) |
| Sample Number  Latin American and Hispanic Ancestry (%) | 1,216 (2.1%) | 1,335 (1.6) |

###

## Table S2: Included BCAC studies

Number of case and control subjects per BCAC study.

| **Study Acronym** | **Country** | **Number Cases** | **Number Controls** |
| --- | --- | --- | --- |
| 2SISTER | USA | 1,118 | 0 |
| ABCS | Netherlands | 347 | 189 |
| ABCTB | Australia | 953 | 375 |
| ACP | Thailand | 753 | 642 |
| AHS | USA | 514 | 1,137 |
| BBCC | Germany | 411 | 253 |
| BBCS | UK | 122 | 442 |
| BCFR-NY | USA | 454 | 27 |
| BCFR-PA | USA | 139 | 0 |
| BCFR-UTAH | USA | 102 | 0 |
| BCINIS | Israel | 1,437 | 724 |
| BREOGAN | Spain | 1,377 | 725 |
| BSUCH | Germany | 277 | 168 |
| CAMA | Mexico | 707 | 657 |
| CBCS | Canada | 335 | 170 |
| CCGP | Greece | 672 | 332 |
| CECILE | France | 306 | 159 |
| CGPS | Denmark | 1,411 | 716 |
| COLBCCC | Colombia | 628 | 559 |
| CPSII | USA | 3,053 | 3,028 |
| CTS | USA | 1,156 | 610 |
| DIETCOMPLYF | UK | 711 | 0 |
| EPIC | France | 433 | 370 |
| EPIC | Germany | 661 | 650 |
| EPIC | Greece | 182 | 180 |
| EPIC | Italy | 822 | 788 |
| EPIC | Netherlands | 709 | 676 |
| EPIC | Spain | 337 | 311 |
| EPIC | UK | 703 | 669 |
| ESTHER | Germany | 296 | 187 |
| FHRISK | UK | 146 | 0 |
| GC-HBOC | Germany | 3,634 | 1,593 |
| GENICA | Germany | 460 | 284 |
| GEPARSIXTO | Germany | 387 | 0 |
| GESBC | Germany | 358 | 181 |
| HABCS | Germany | 928 | 866 |
| HCSC | Spain | 426 | 0 |
| HEBCS | Finland | 281 | 177 |
| HERPACC | Japan | 282 | 282 |
| HKBCS | Hong Kong | 564 | 453 |
| HMBCS | Belarus | 212 | 249 |
| HUBCS | Russia | 211 | 120 |
| KARBAC | Sweden | 503 | 0 |
| KARMA | Sweden | 2,645 | 6,026 |
| KBCP | Finland | 556 | 245 |
| KOHBRA | Korea | 1,464 | 665 |
| LMBC | Belgium | 805 | 1,268 |
| MABCS | Macedonia | 90 | 92 |
| MARIE | Germany | 512 | 289 |
| MBCSG | Italy | 788 | 366 |
| MCBCS | USA | 926 | 221 |
| MEC | USA | 1,317 | 1,450 |
| MISS | Sweden | 701 | 1,545 |
| MMHS | USA | 384 | 1,635 |
| MSKCC | USA | 138 | 0 |
| MYBRCA | Malaysia | 844 | 1,257 |
| NBCS | Norway | 1,283 | 0 |
| NBHS | USA | 887 | 795 |
| NC-BCFR | USA | 1,474 | 207 |
| NCBCS | USA | 4,430 | 1,613 |
| NGOBCS | Japan | 369 | 366 |
| NHS | USA | 1,590 | 1,804 |
| NHS2 | USA | 1,607 | 1,905 |
| ORIGO | Netherlands | 1,053 | 0 |
| PBCS | Poland | 1,931 | 2,045 |
| PKARMA | Sweden | 834 | 48 |
| PLCO | USA | 2,330 | 2,666 |
| POSH | UK | 1,088 | 0 |
| PREFACE | Germany | 2,989 | 0 |
| PROCAS | UK | 647 | 1,656 |
| SBCGS | China | 832 | 932 |
| SEARCH | UK | 4,057 | 2,673 |
| SEBCS | Korea | 1,101 | 1,106 |
| SGBCC | Singapore | 895 | 703 |
| SISTER | USA | 2,167 | 1,731 |
| SKKDKFZS | Germany | 1,095 | 0 |
| SMC | Sweden | 1,509 | 704 |
| SUCCESSB | Germany | 440 | 0 |
| SUCCESSC | Germany | 2,836 | 0 |
| TNBCC | Germany | 245 | 0 |

##

## Table S3: Number of genes analysed per cohort

For each cohort, only genes were included in the analysis which contained at least 3 SNPs and equal to or less than 5000 SNPs.

| **Cohort** | **Number analysed genes** | **Total number genes** |
| --- | --- | --- |
| african | 18,389 | 18,996 |
| asian | 17,686 | 18,996 |
| eur01A | 16,717 | 18,996 |
| eur01B | 16,762 | 18,996 |
| eur01C | 17,902 | 18,996 |
| eur01D | 17,739 | 18,996 |
| eur02A | 17,464 | 18,996 |
| eur02B | 17,942 | 18,996 |
| eur03A | 16,571 | 18,996 |
| eur03B | 17,887 | 18,996 |
| eur04 | 17,566 | 18,996 |
| eur05A | 17,955 | 18,996 |
| eur05B | 17,938 | 18,996 |
| eur05C | 17,906 | 18,996 |
| latin american and hispanic | 18,497 | 18,996 |

## Table S4: Genomic inflation in burden analysis

Based on burden analysis P-values for individual genes in each cohort, genomic inflation factor ƛ was calculated as well as ƛ1000 which accounts for sample size. Cohorts with increased genomic inflation factor, cohorts “eur02B” and “latin american and hispanic”, are highlighted in grey.

| **Cohort** | **Number Cases** | **Number Controls** | **Lambda** | **Lambda 1000** |
| --- | --- | --- | --- | --- |
| african | 3,678 | 2,056 | 1.03 | 1.01 |
| asian | 8,390 | 6,831 | 1.08 | 1.01 |
| eur01A | 6,350 | 4,802 | 0.96 | 0.99 |
| eur01B | 5,187 | 4,238 | 1.17 | 1.04 |
| eur01C | 5,982 | 5,422 | 1.14 | 1.02 |
| eur01D | 5,012 | 4,552 | 1.09 | 1.02 |
| eur02A | 7,576 | 2,124 | 1.07 | 1.02 |
| eur02B | 7,953 | 2,347 | 2.16 | 1.32 |
| eur03A | 5,049 | 3,472 | 1.05 | 1.01 |
| eur03B | 4,057 | 3,647 | 1.03 | 1.01 |
| eur04 | 6,192 | 8,323 | 1.12 | 1.02 |
| eur05A | 5,698 | 3,869 | 1.16 | 1.03 |
| eur05B | 5,298 | 3,532 | 1.04 | 1.01 |
| eur05C | 5,626 | 3,366 | 1.15 | 1.04 |
| latin american and hispanic | 1,335 | 1,216 | 1.17 | 1.14 |

## Table S5: Model covariates per cohort

Covariates included in MONSTER gene association models for individual cohorts. In some cohorts, inclusion of recruitment study and/or study country covariates would have correlated significantly with phenotype outcome and, thus, covariates were not included in the affected models.

| **Cohort** | **Fixed Effect Covariate** | **Random Effect Covariate** |
| --- | --- | --- |
| african | age at examination, recruitment study | kinship matrix |
| asian | age at examination, recruitment study | kinship matrix |
| eur01A | age at examination, recruitment study | kinship matrix |
| eur01B | age at examination, recruitment study | kinship matrix |
| eur01C | age at examination, recruitment study | kinship matrix |
| eur01D | age at examination, recruitment study | kinship matrix |
| eur02A | age at examination | kinship matrix |
| eur02B | age at examination | kinship matrix |
| eur03A | age at examination, recruitment study | kinship matrix |
| eur03B | age at examination, recruitment study | kinship matrix |
| eur04 | age at examination, recruitment study | kinship matrix |
| eur05A | age at examination, study country | kinship matrix |
| eur05B | age at examination, study country | kinship matrix |
| eur05C | age at examination, study country | kinship matrix |
| latin american and hispanic | age at examination, recruitment study | kinship matrix |

###

## Figure S1: QQ-plots of burden analysis

To inspect whether the distribution of burden P-values for individual genes in each cohort follows an approximate normal distribution quantile-quantile plots were created.


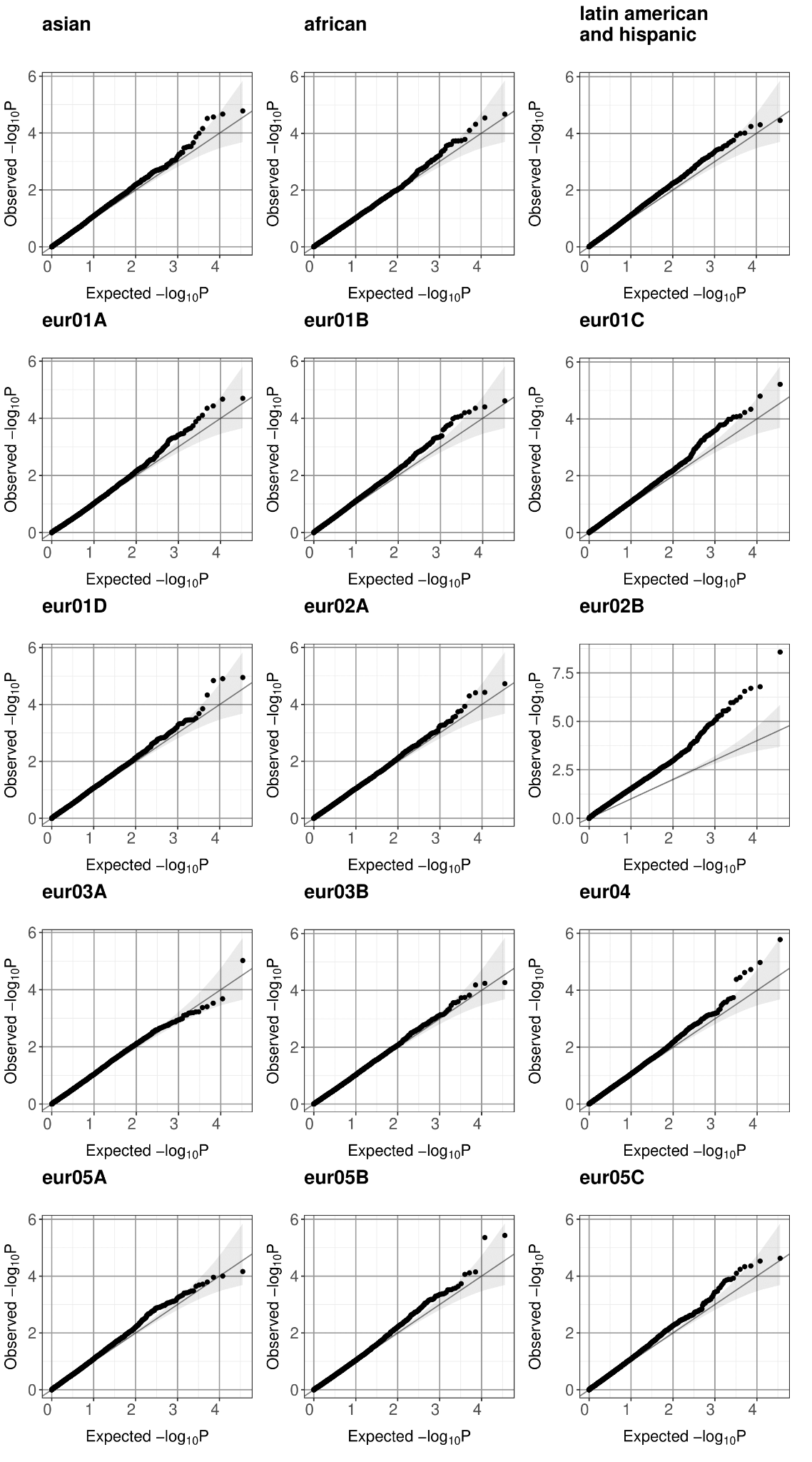


## Figure S2: QQ-plots for meta-analysis

To inspect whether the distribution of burden P-values for individual genes in meta-analysis follows an approximate normal distribution quantile-quantile plots were created.


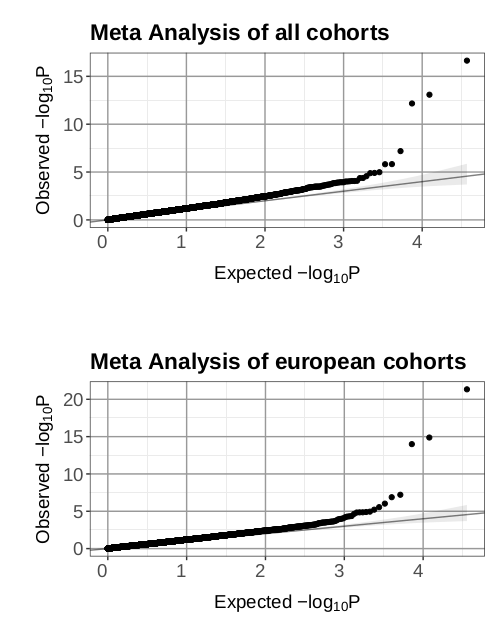


## Figure S3: Regional plots for significantly associated genes

Regional plots of gene regions found to be significantly associated with breast cancer in meta-analysis of European samples.  **A** Depiction of coding regions of all coding genes (data retrieved from Ensembl biomart hg38) in chromosomal region. Highlighted in blue are significantly associated genes of interest. **B** Variants included in MONSTER aggregation test are plotted according to their chromosomal position and variant weight using Phred-scaled EigenPC [(2)](https://paperpile.com/c/EH80kH/FoKsD) pathogenicity scores. Highlighted in blue are variants only observed in samples of diverse ancestry. **C** Single marker association results as reported by genome-wide association study in BCAC samples [(1)](https://paperpile.com/c/EH80kH/yI6Dc) are displayed with blue solid line giving P-value for meta-analysis of all cohorts for gene of interest in this study and blue dashed line giving unadjusted P-value for all European meta-analysis.

### FAM72B


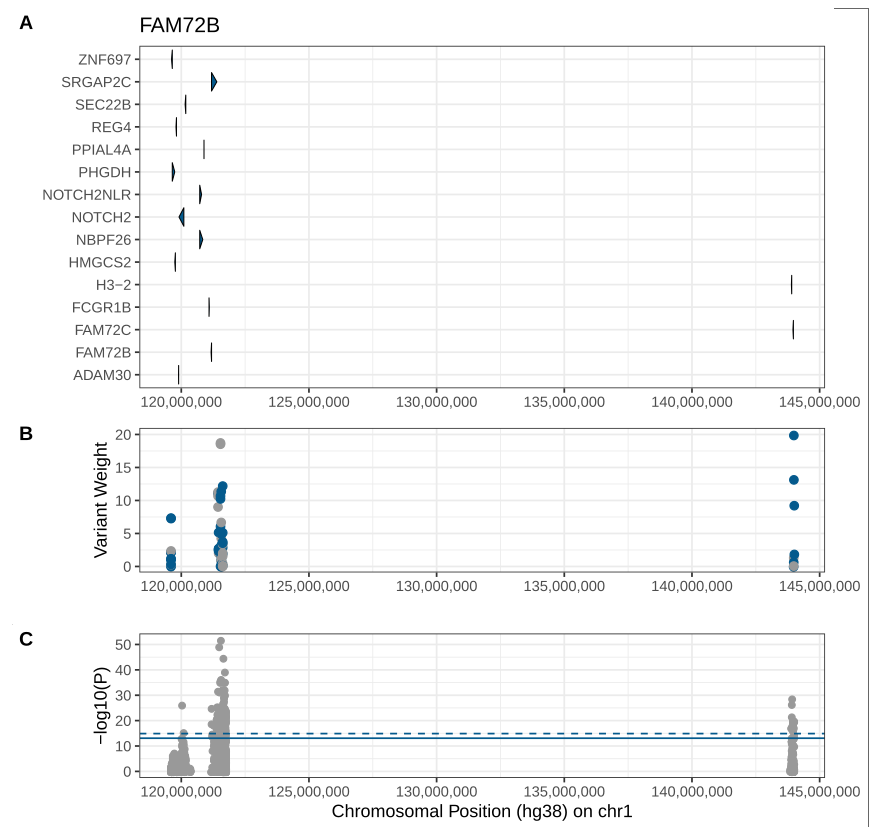


### SRGAP2C


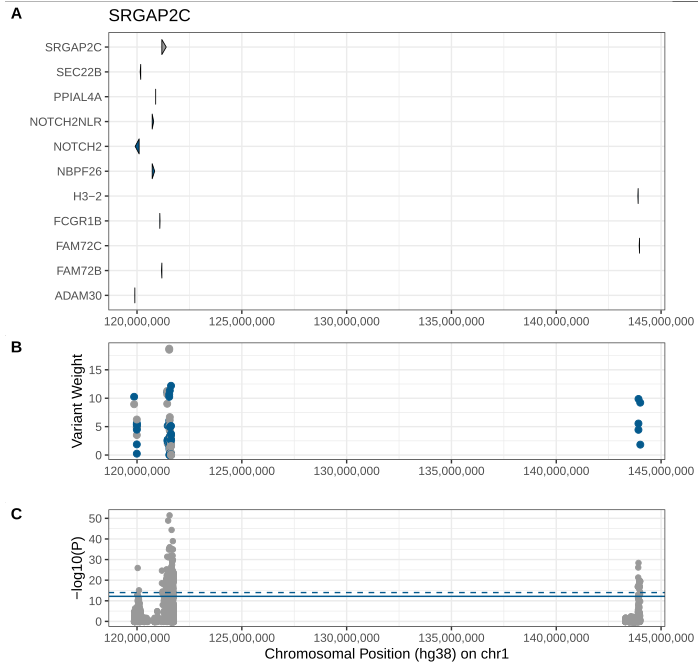


### AC058822.1

#
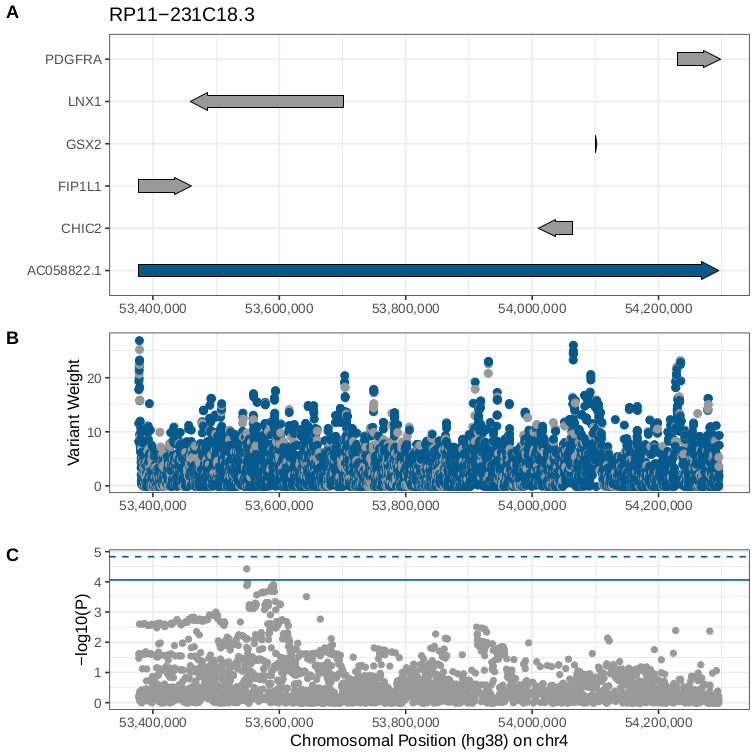


### ABRAXAS1


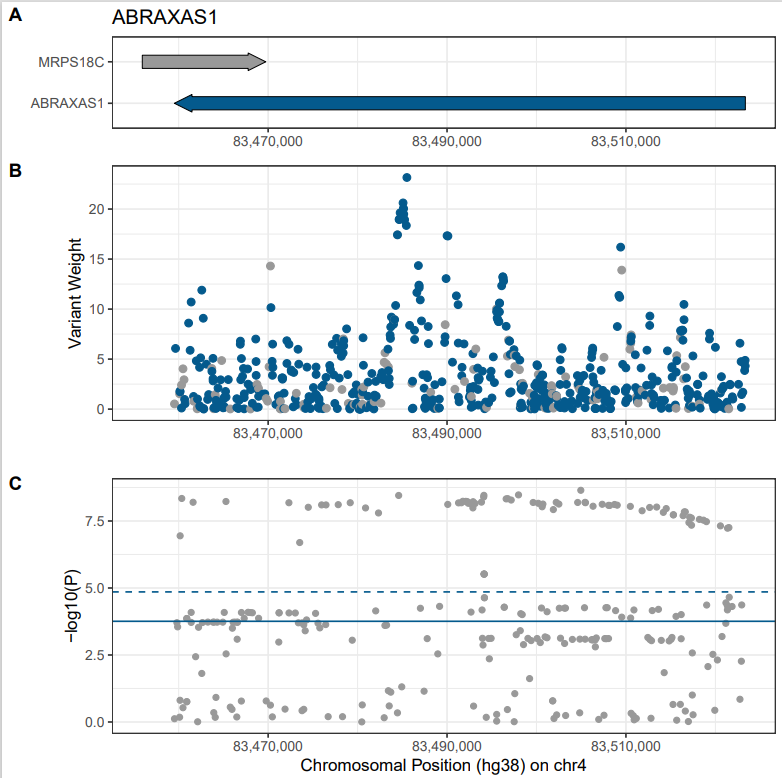


### MAP3K1

#
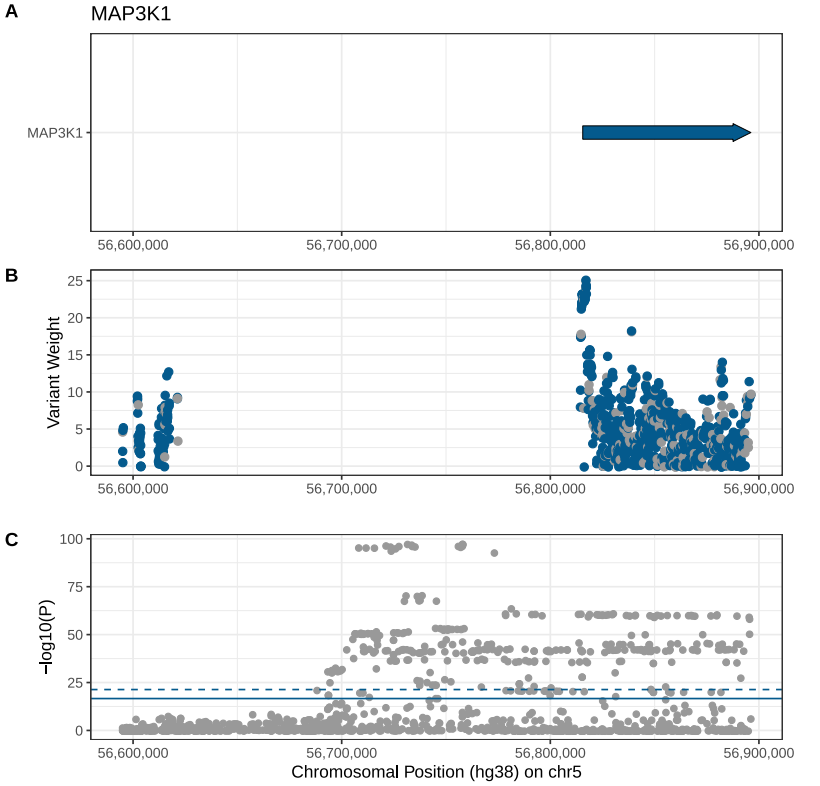


### MIER3


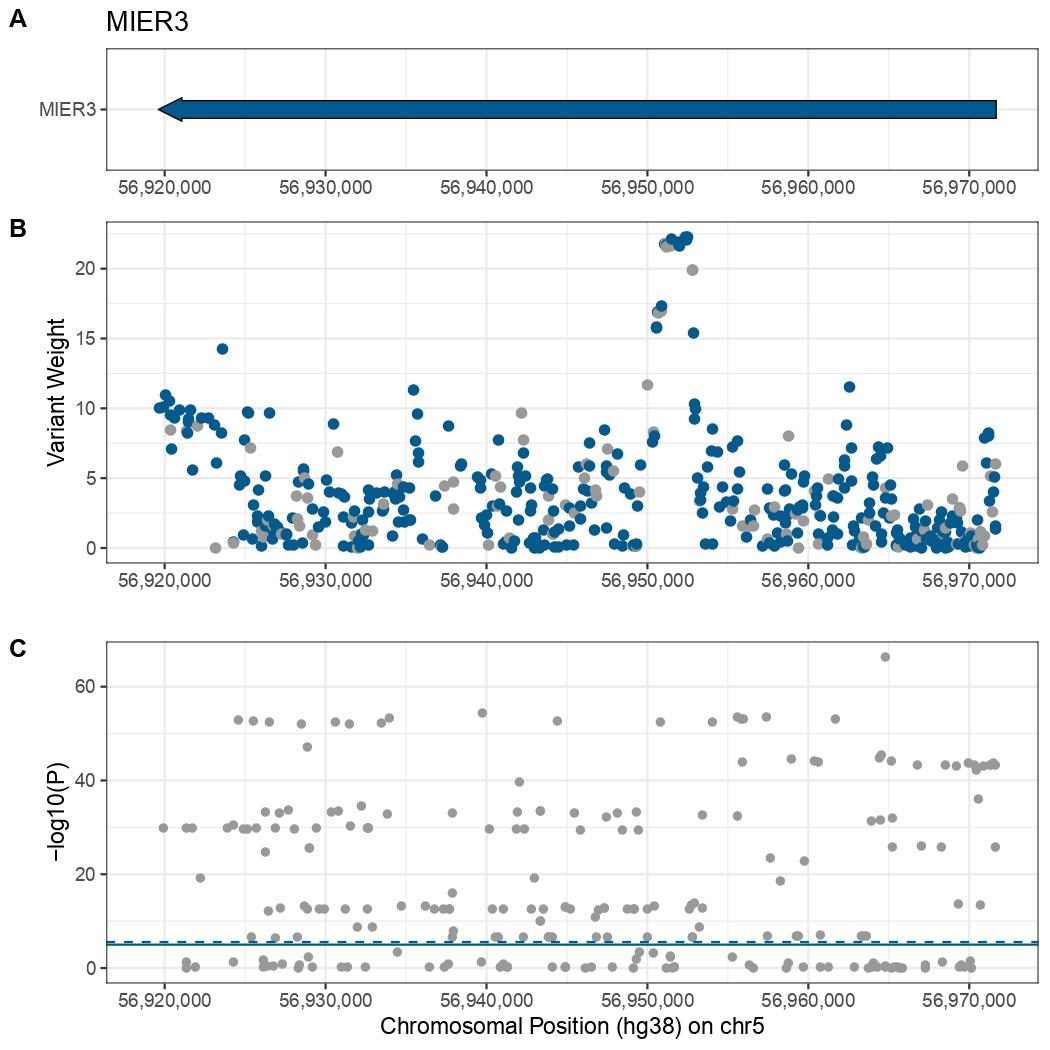


### BTN2A1


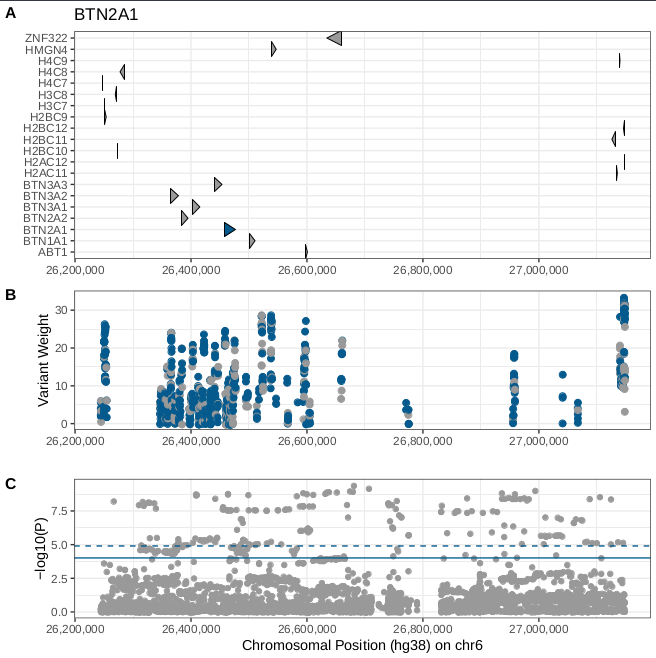


### FGFR2


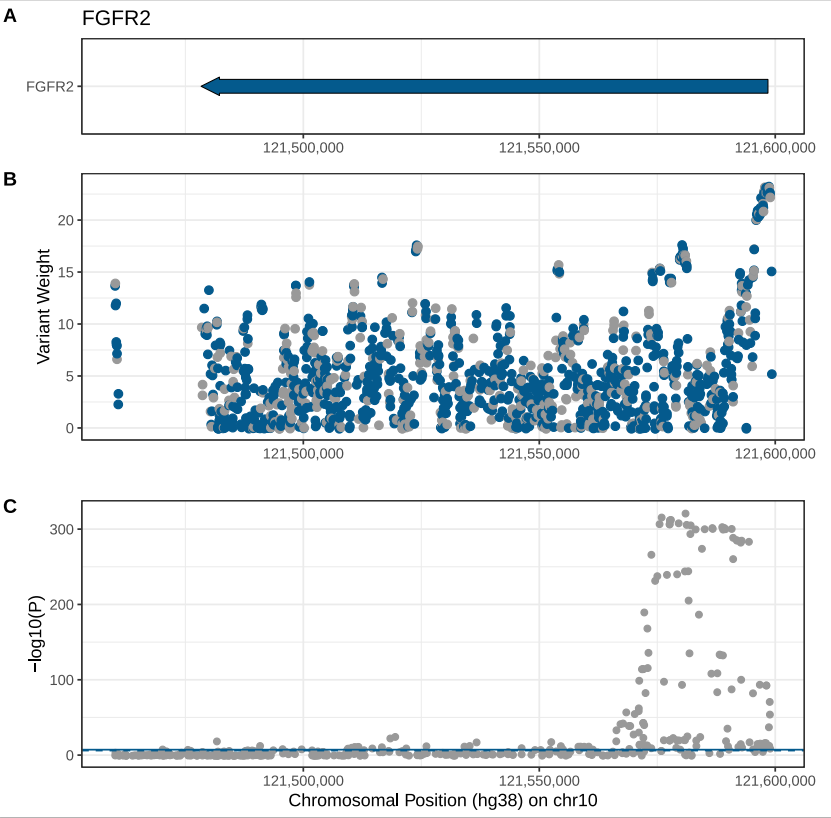


### TNNT3


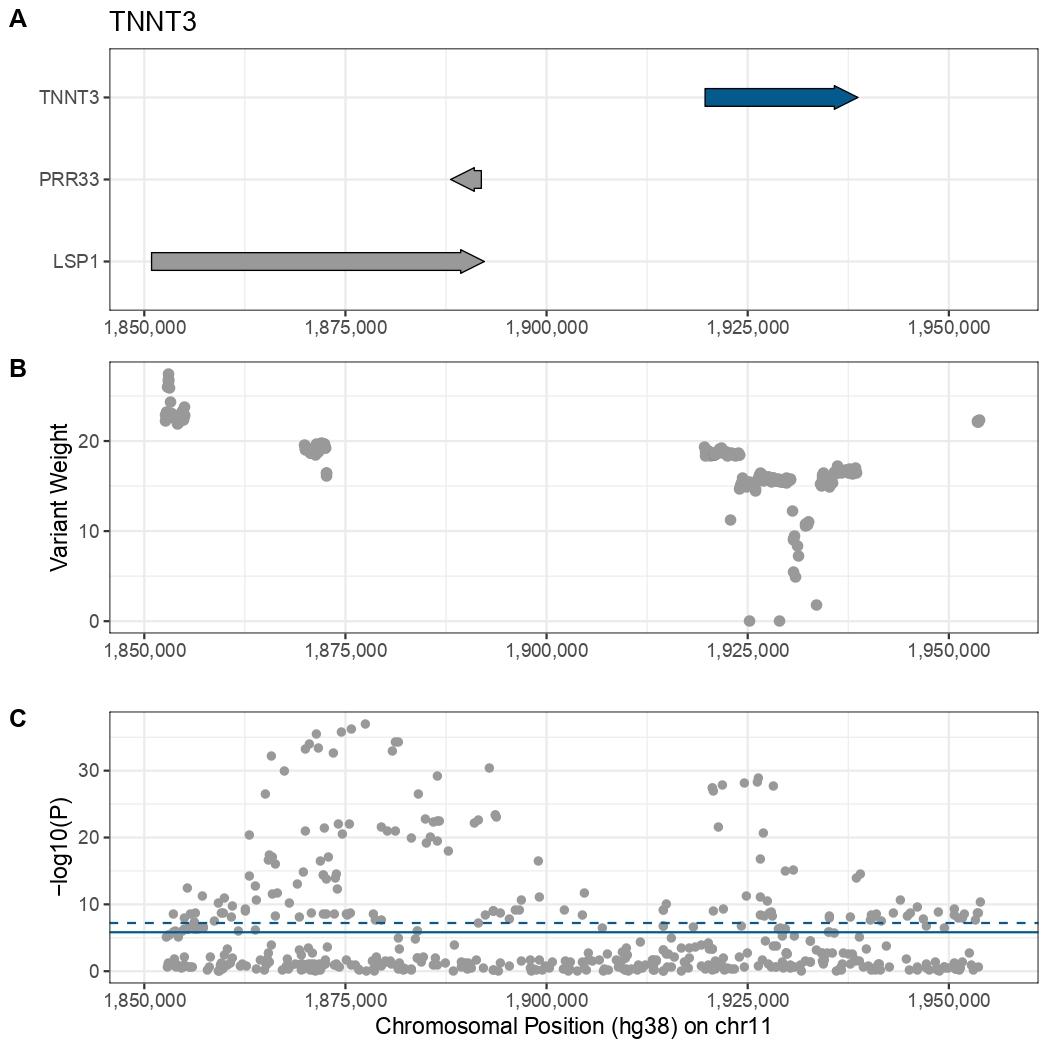


=

### LSP1


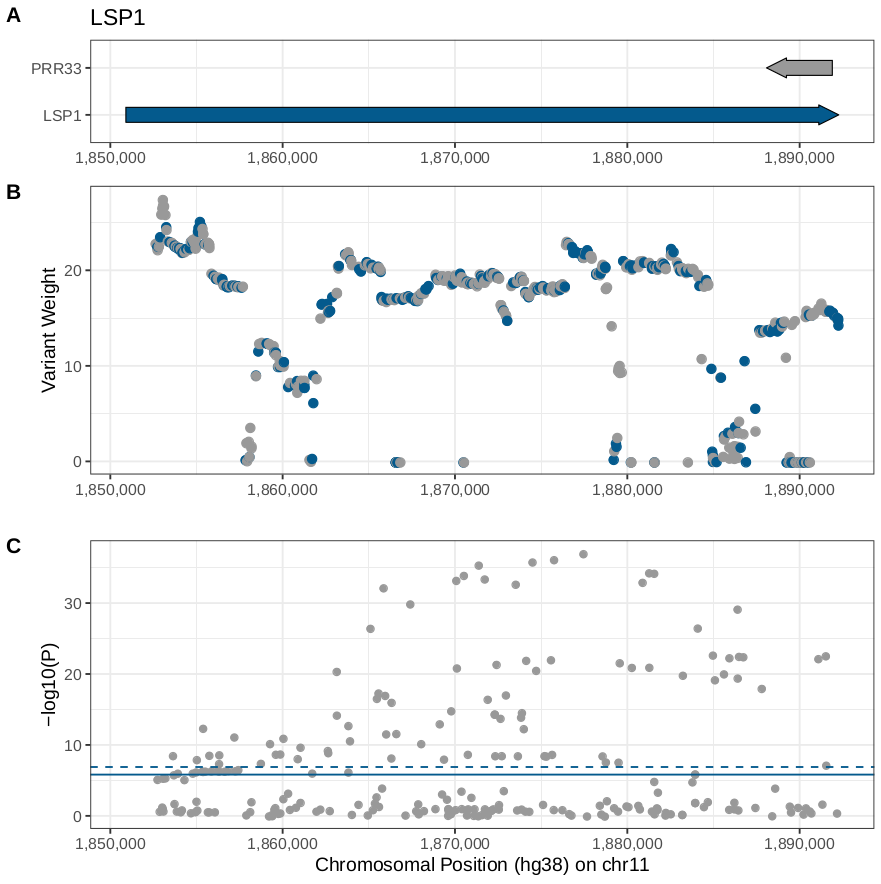


### LYPD5


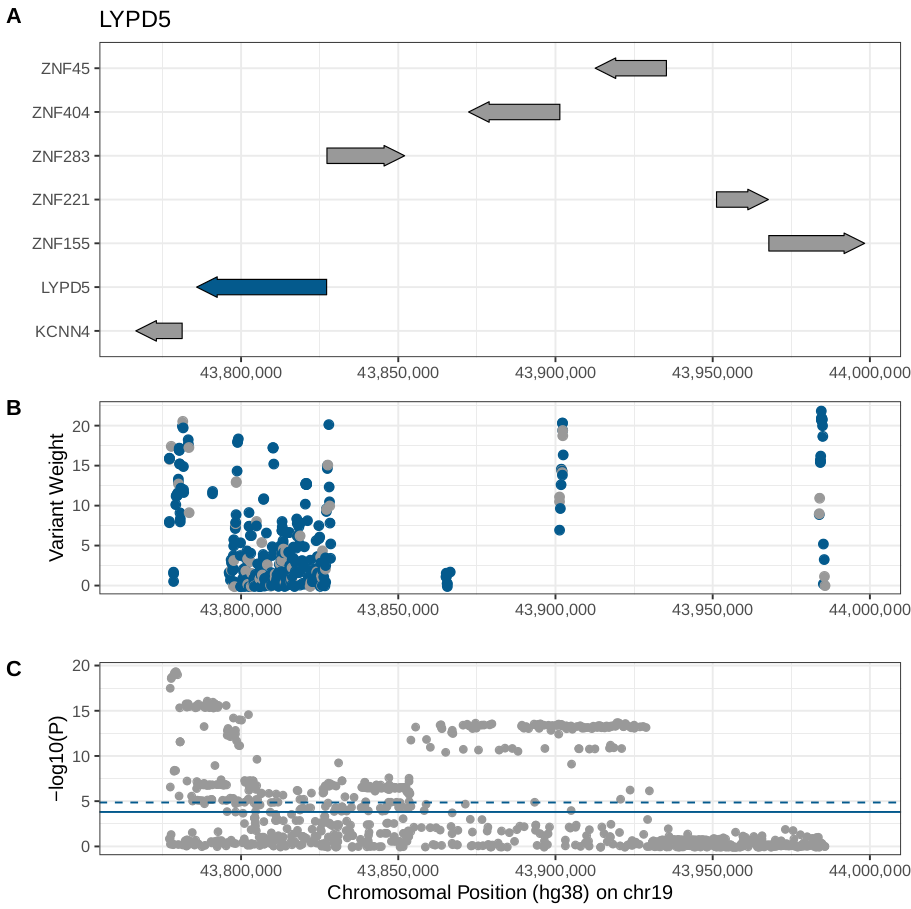


### KCNN4


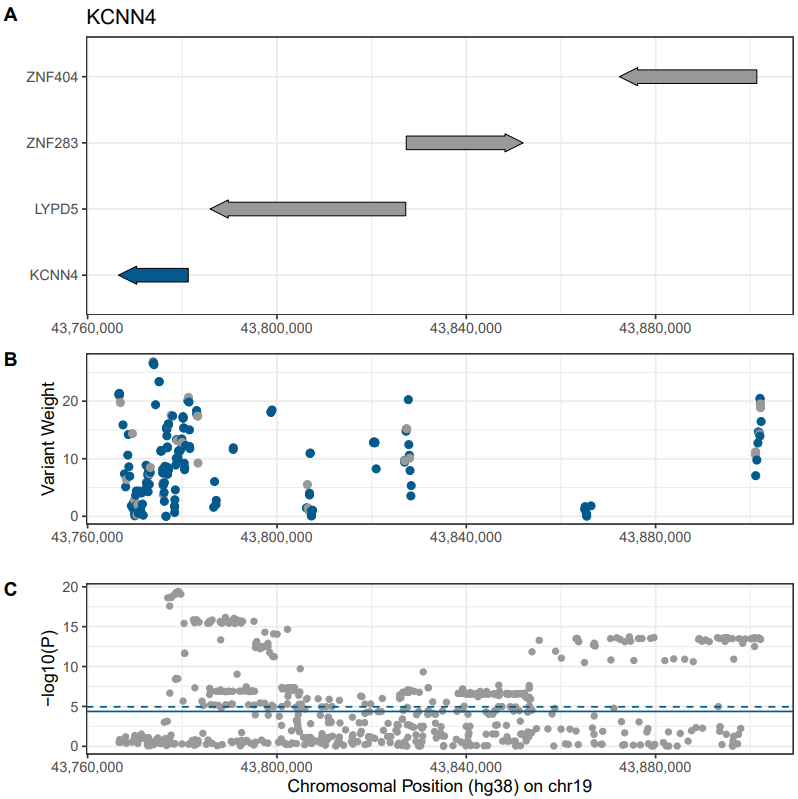


### ZNF404


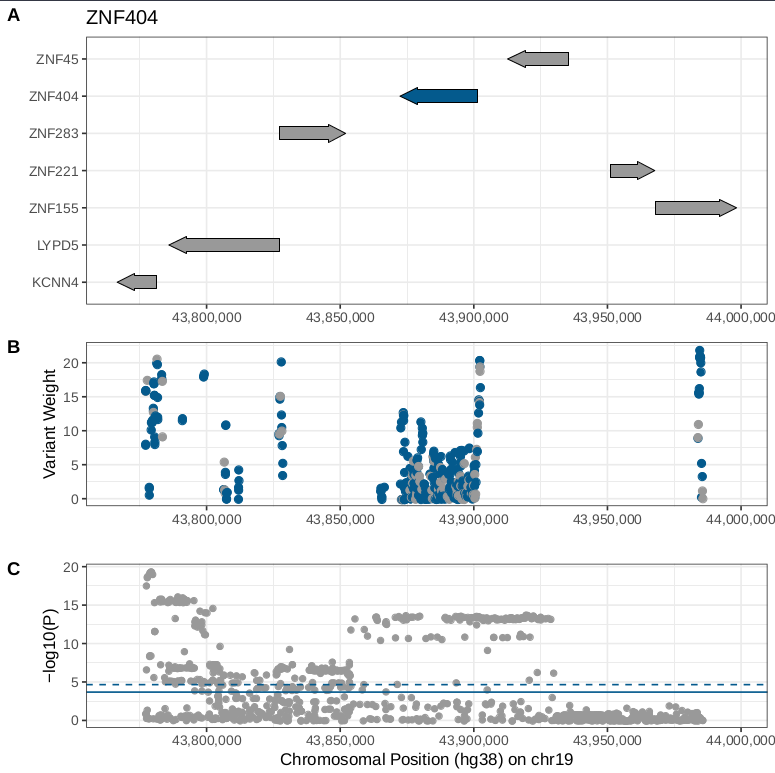


## Table S6: All Cohort Meta-Analysis

Gene-based associations with breast cancer were combined using Stouffer meta-analysis in either an all-European meta-analysis (Number cohorts = 12) or an all-cohort meta-analysis (Number cohorts = 15). For each meta-analysis unadjusted and FDR multiple testing adjusted P-values are reported. Significant associations in the all-cohort meta-analysis (adjusted P-Value<0.05) are highlighted in grey. Genes with overlapping genetic regions in the aggregation analysis are combined as a single genetic locus.

|  |  |  | **all-European Meta-Analysis** | | **all-Cohort Meta-Analysis** | |
| --- | --- | --- | --- | --- | --- | --- |
| **Locus (hg38)** | **Stable Gene ID** | **Gene** | **unadjusted**  **P-value** | **adjusted P-value** | **unadjusted**  **P-value** | **adjusted P-value** |
| chr5:56,815,574-56,971,675 | ENSG00000095015 | MAP3K1 | 4.61E-22 | 8.28E-18 | 2.28E-17 | 4.22E-13 |
|  | ENSG00000155545 | MIER3 | 2.81E-06 | 7.22E-03 | 1.04E-05 | 2.69E-02 |
| chr1:121,167,646-121,392,822 | ENSG00000188610 | FAM72B | 1.32E-15 | 1.19E-11 | 8.26E-14 | 7.66E-10 |
|  | ENSG00000171943 | SRGAP2C | 1.01E-14 | 6.07E-11 | 6.84E-13 | 4.23E-09 |
| chr10:121,478,334-121,598,458 | ENSG00000066468 | FGFR2 | 9.48E-07 | 2.84E-03 | 6.52E-08 | 3.02E-04 |
| chr11:1,852,970-1,938,706 | ENSG00000130595 | TNNT3 | 6.17E-08 | 2.77E-04 | 1.48E-06 | 4.72E-03 |
|  | ENSG00000130592 | LSP1 | 1.31E-07 | 4.70E-04 | 1.53E-06 | 4.72E-03 |
| chr12:49,636,499-49,708,165 | ENSG00000161791 | FMNL3 | 6.11E-06 | 1.37E-02 | 1.24E-05 | 2.69E-02 |
| chr6:151,656,734-152,129,452 | ENSG00000091831 | ESR1 | 2.77E-04 | 1.67E-01 | 1.31E-05 | 2.69E-02 |
| chr19:43,766,533-43,901,385 | ENSG00000104783 | KCNN4 | 1.12E-05 | 2.03E-02 | 4.27E-05 | 6.60E-02 |
|  | ENSG00000159871 | LYPD5 | 1.39E-05 | 2.03E-02 | 1.54E-04 | 1.10E-01 |
|  | ENSG00000176222 | ZNF404 | 2.23E-05 | 2.86E-02 | 2.09E-04 | 1.29E-01 |
| chr4:53,377,839-54,295,272 | ENSG00000282278 | AC058822.1 | 1.47E-05 | 2.03E-02 | 8.64E-05 | 1.01E-01 |
| chr4:83,459,517-83,523,348 | ENSG00000163322 | ABRAXAS1 | 1.40E-05 | 2.03E-02 | 1.75E-04 | 1.20E-01 |
| chr6:26,457,904-26,476,621 | ENSG00000112763 | BTN2A1 | 1.26E-05 | 2.03E-02 | 9.75E-05 | 1.01E-01 |

## Table S7: Suggestive Associations in Diverse Ancestries

Suggestive gene associations in individual cohorts of diverse ancestry, African (n=5,784), Asian (n=15,321) and Latin American and Hispanic (n=2,551) cohort, were identified with unadjusted P-values below 1x10^-4^.

Regions of associated genes with an additional flanking region of 100kB were queried in published GWAS summary statistics from Michailidou *et al.* [(1)](https://paperpile.com/c/EH80kH/yI6Dc/?noauthor=1) for overlap with previously reported associated variants, defined as a minimum unadjusted P-value in Michailidou (2017) study below 5x10^-8^.

| **Locus (hg38)** | **Stable Gene ID** | **Gene** | **Cohort with**  **association** | **unadjusted**  **P-value** | **Michailidou (2017)**  **GWAS association** | **Minimum unadjusted**  **P-value in**  **Michailidou (2017) study** |
| --- | --- | --- | --- | --- | --- | --- |
| chr9:122,237,624-122,331,343 | ENSG00000119446 | RBM18 | Asian | 0.0000274 | No | 0.00439 |
|  | ENSG00000148187 | MRRF | Asian | 0.0000167 | No | 0.00439 |
| chr3:105,655,461-105,869,552 | ENSG00000114423 | CBLB | African | 0.0000211 | No | 0.00308 |
| chr11:89,797,655-89,808,575 | ENSG00000168930 | TRIM49 | Asian | 0.0000217 | No | 0.00295 |
| chr3:15,450,133-15,521,751 | ENSG00000206561 | COLQ | African | 0.0000286 | No | 0.00475 |
| chr19:51,311,848-51,330,354 | ENSG00000142549 | IGLON5 | Asian | 0.0000307 | No | 0.00159 |
| chr3:15,667,236-15,859,771 | ENSG00000206560 | ANKRD28 | African | 0.0000478 | No | 0.00475 |
| chr19:51,246,348-51,269,330 | ENSG00000179213 | SIGLECL1 | Asian | 0.0000695 | No | 0.0045 |
| chr10:124,619,292-124,744,269 | ENSG00000189319 | FAM53B | African | 0.0000786 | No | 0.000016 |

##

## Table S8: Prior Evidence for Suggestive Associations in Diverse Ancestries

Genes with suggestive association significance (unadjusted P-value <0.05) in individual diverse cohorts were queried in public databases for evidence of causal effects in breast cancer or any cancer. Genes with some level of causal evidence are highlighted in grey. Please see Table 2 for detailed table description.

|  |  | **Causal evidence in breast cancer** | | | **Causal evidence in any cancer** | | |  |
| --- | --- | --- | --- | --- | --- | --- | --- | --- |
| **Locus (hg38)** | **Gene** | **ClinVar** | **Genetics Home**  **Reference** | **Malacards**  **Score>1** | **ClinVar** | **Genetics Home**  **Reference** | **Malacards**  **Score>1** | **Cancer Gene Census**  **[TIER1;TIER2;NO]** |
| chr19:51,246,348-51,269,330 | SIGLECL1 | NO | NO | NO | NO | NO | NO | NO |
| chr19:51,311,848-51,330,354 | IGLON5 | NO | NO | NO | NO | NO | NO | NO |
| chr9:122,237,624-122,264,839 | RBM18 | NO | NO | NO | NO | NO | NO | NO |
| chr9:122,264,603-122,331,343 | MRRF | NO | NO | NO | NO | NO | NO | NO |
| chr11:89,797,655-89,808,575 | TRIM49 | NO | NO | NO | NO | NO | NO | NO |
| chr3:15,450,133-15,521,751 | COLQ | NO | NO | NO | NO | NO | NO | NO |
| chr3:105,655,461-105,869,552 | **CBLB** | NO | NO | **YES** | NO | NO | **YES** | NO |
| chr3:15,667,236-15,859,771 | **ANKRD28** | NO | NO | NO | NO | NO | **YES** | NO |
| chr10:124,619,292-124,744,269 | **FAM53B** | NO | NO | NO | NO | NO | **YES** | NO |

##

## Figure S4: Regional plot for gene *ESR1*

Please see Figure S3 for detailed plot description. Plotted in panel **C** are GWAS summary statistics for the genetic region in Michailidou *et al.* [(1)](https://paperpile.com/c/EH80kH/yI6Dc/?noauthor=1) GWAS based European ancestry samples in BCAC. Multiple variants were found to be nominally associated with breast cancer in this region. Unadjusted P-value for gene *ESR1* in the all-European (P-value: 2.77 x 10^-4^) and the all-cohort meta-analysis (P-value: 1.31 x 10^-5^) are included as solid and dashed horizontal lines, respectively.


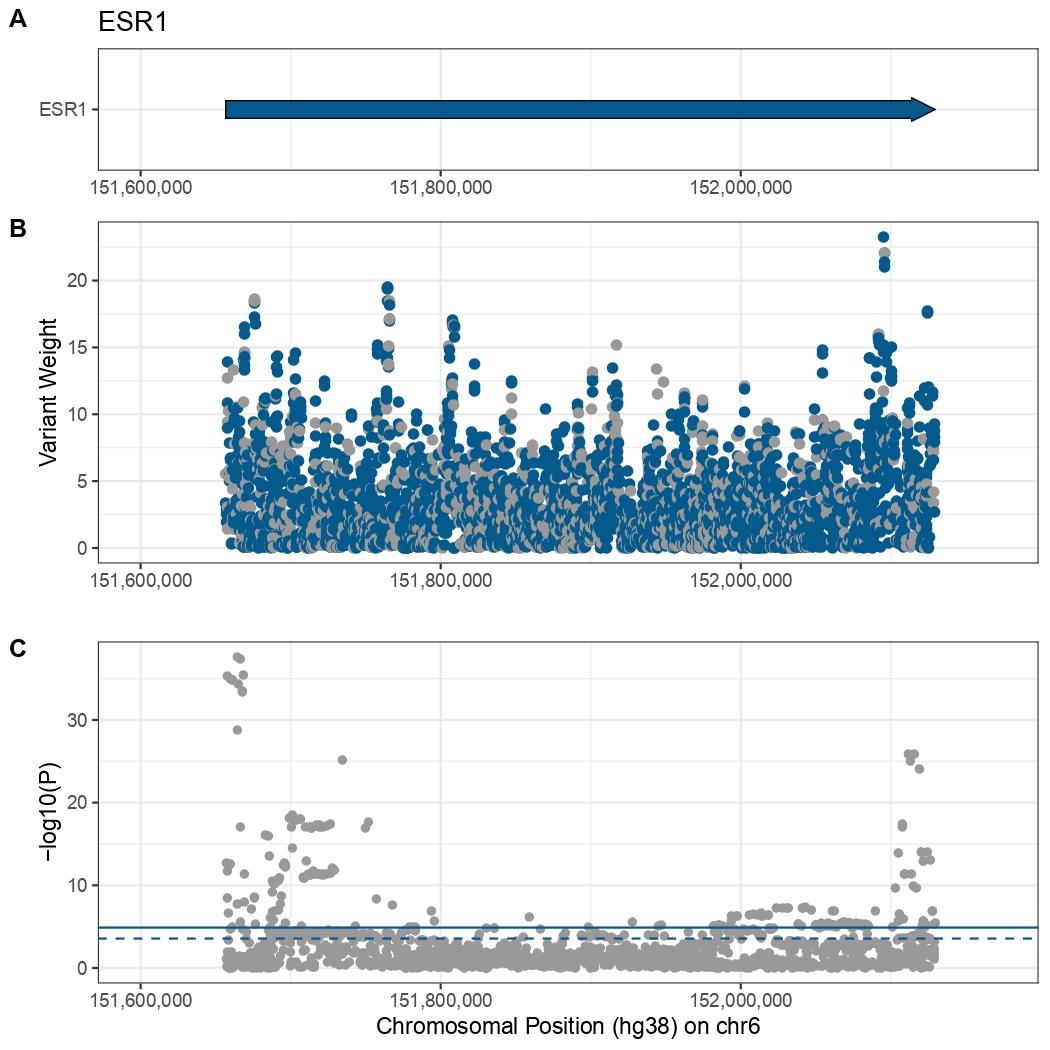


## Figure S5: Regional plot for gene *CBLB*

Please see Figure S3 for detailed plot description. Gene *CBLB* was found with suggestive association significance (unadjusted P-Value: 2.11x10^-5^, green horizontal line in Panel C), in African cohort. Panel **C**  reveals no significantly associated variants in Michailidou *et al.* [(1)](https://paperpile.com/c/EH80kH/yI6Dc/?noauthor=1) GWAS in this genetic region.

Unadjusted P-value for gene *CBLB* in the all-European (P-value: 0.230) and the all-cohort meta-analysis (P-value: 0.031) are included as solid and dashed horizontal lines, respectively.


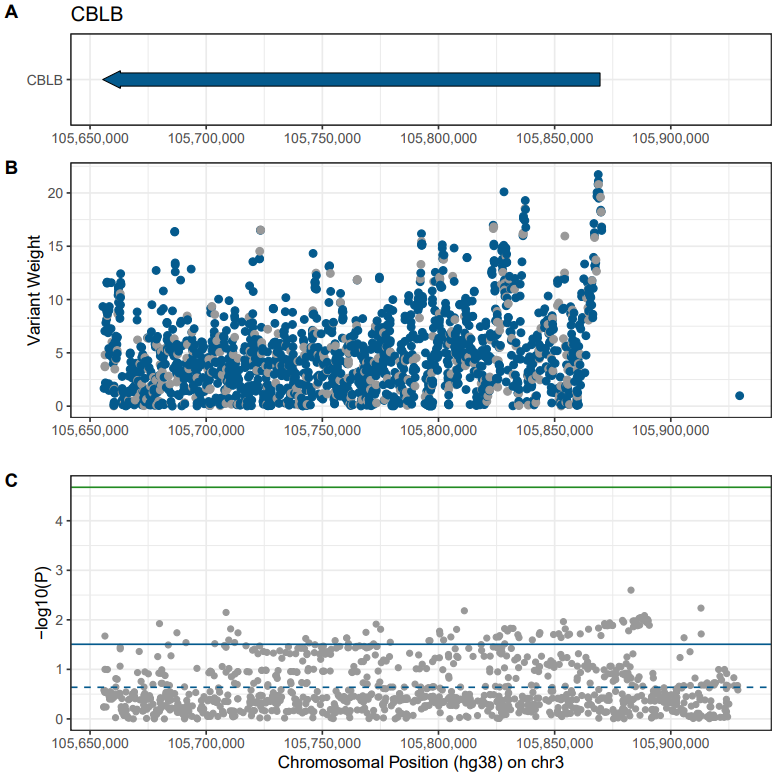


##

## Table S9: Ethics committees that provided approval for the contributing studies

| **Study** | **Acronym** | **Country** | **Approval Committee(s)** |
| --- | --- | --- | --- |
| The Two Sister Study | 2SISTER | USA | Institutional Review Board of the National Institute of Environmental Health Sciences; NIH and the Copernicus Group Independent Review Board |
| Amsterdam Breast Cancer Study | ABCS | Netherlands | Leiden University Medical Center (LUMC) Commissie Medische Ethiek; Protocol Toetsingscommissie van Het Nederlands Kanker Instituut-Antoni van Leeuwenhoek Ziekenhuis |
| Australian Breast Cancer Tissue Bank | ABCTB | Australia | Sydney Local Health District (RPA Zone) Research Ethics and Governance Office |
| Asia Cancer Program | ACP | Thailand | Khon Kaen University Ethics Committee for Human Research; Ethics Committee of National Cancer Institute Thailand; Prince of Songkla University Faculty of Medicine Ethics Committee; HRH Princess Maha Chakri Sirindhorn Medical Centre (MSMC) Ethics Committee |
| Agricultural Health Study | AHS | USA | National Institute of Health (NIH) Population Sciences IRB |
| Bavarian Breast Cancer Cases and Controls | BBCC | Germany | Friedrich-Alexander-Universitat Erlangen-Nurnberg Medizinische Fakultat Ethik-Commission |
| British Breast Cancer Study | BBCS | UK | South East Multi-Centre Research Ethics Committee |
| New York Breast Cancer Family Registry | BCFR-NY | USA | Columbia University Institutional Review Board |
| Philadelphia Breast Cancer Family Registry | BCFR-PA | USA | Institutional Review Board Fox Chase Cancer Center |
| Utah Breast Cancer Family Registry | BCFR-UTAH | USA | University of Utah Institutional Review Board |
| Breast Cancer In Northern Israel Study | BCINIS | Israel | Carmel Medical Center |
| Breast Oncology Galicia Network | BREOGAN | Spain | Comité Autonómico de Ética de la Investigación de Galicia |
| Breast Cancer Study of the University Clinic Heidelberg | BSUCH | Germany | Ethikkommission Medizinische Fakultat Heidelberg, University of Heidelberg |
| CAMA-Mexican Breast Cancer Study | CAMA | Mexico | IRB of the National Institute of Public Health (INSP) of Mexico; UCSF Committee on Human Research |
| Canadian Breast Cancer Study | CBCS | Canada | University of British Columbia - BC Cancer Research Ethics Board; Queen’s University Health Sciences and Affiliated Teaching Hospitals Research Human Ethics Board (HSREB) |
| Crete Cancer Genetics Program | CCGP | Greece | Epistimoniko Symvoulio (Scientific Council of the University General hospital of Heraklion) |
| CECILE Breast Cancer Study | CECILE | France | Comité Consultatif de Protection des Personnes dans la Recherche Biomédicale de Bicêtre (Le Kremlin-Bicêtre FR-94270) |
| Copenhagen General Population Study | CGPS | Denmark | Kobenhavns Amt den Videnskabsetiske Komite (Scientific ethical committee, Copenhagen County) |
| Colombian Breast Cancer Case-Control Study | COLBCCC | Colombia | Comite de Investigaciones y Etica Pontificia Universidad Javeriana Facultad de Medicina |
| Cancer Prevention Study-II Nutrition Cohort | CPSII | USA | Emory University Institutional Review Board |
| California Teachers Study | CTS | USA | UC Irvine: Office of Research Institutional Review Board |
| DietCompLyf Breast Cancer Survival Study | DIETCOMPLYF | UK | MREC Committee A and NRES Committee London –Bentham |
| European Prospective Investigation Into Cancer and Nutrition | EPIC | Various within EU | Institutional Review Board of the International Agency for Research on Cancer, Lyon, France; Ethics Commission of the Faculty of Medicine of the University of Heidelberg |
| ESTHER Breast Cancer Study | ESTHER | Germany | Ruprecht-Karls-Universitat Medizinische Fakultat Heidelberg Ethikkommission |
| Family History Risk Study | FHRISK | UK | NRES Committee North West - Greater Manchester Central |
| German Consortium for Hereditary Breast & Ovarian Cancer | GC-HBOC | Germany | Ethik-Kommission der Medizinischen Fakultat der Universitat zu Koln |
| Gene Environment Interaction and Breast Cancer in Germany | GENICA | Germany | Ethikkommission Rheinische Friedrich-Wilhels-Universität Bonn |
| A randomized phase II trial investigating the addition of carboplatin to neoadjuvant therapy for triple-negative and HER2-positive early breast cancer | GEPARSIXTO | Germany | Ethikkommission der Ärztkammer Nordrhein |
| Genetic Epidemiology Study of Breast Cancer by Age 50 | GESBC | Germany | Medizinische Fakultat Heidelberg Ethikkommission |
| Hannover Breast Cancer Study | HABCS | Germany | Medizinische Hochschule Hannover Ethik-Kommission |
| Hospital Clinico San Carlos | HCSC | Spain | Hospital Clínico San Carlos Ethical Committee |
| Helsinki Breast Cancer Study | HEBCS | Finland | Helsingin ja uudenmaan sairaanhoitopiiri (Helsinki University Hospital Ethics Committee) |
| Hospital-based Epidemiologic Research Program at Aichi Cancer Center | HERPACC | Japan | Aichi Cancer Center Ethics Committee |
| Hong Kong Breast Cancer Study | HKBCS | Hong Kong | Institutional Review Board of the University of Hong Kong/Hospital Authority Hong Kong Cluster (HKU/HA HKW IRB) |
| Hannover-Minsk Breast Cancer Study | HMBCS | Belarus | Medizinische Hochschule Hannover Ethik-Kommission |
| Hannover-Ufa Breast Cancer Study | HUBCS | Russia | Ethical Committee of Institute of Biochemistry and Genetics, Ufa Scientific Center of Russian Academy of Sciences |
| Karolinska Breast Cancer Study | KARBAC | Sweden | Regionala Etikprovningsnamnden i Stockholm (Regional Ethical Review Board in Stockholm) |
| Karolinska Mammography Project for Risk Prediction of Breast Cancer - Cohort Study | KARMA | Sweden | Regionala Etikprovningsnamnden i Stockholm (Regional Ethical Review Board in Stockholm) |
| Kuopio Breast Cancer Project | KBCP | Finland | Pohjois-Savon Sairraanhoitopiirin Kuntayhtyma Tutkimuseettinen Toimikunta |
| Korean Hereditary Breast Cancer Study | KOHBRA | Korea | Seoul National University College of Medicine/Seoul National University Hospital Institutional Review Board (SNUCM/SNUH IRB) |
| Leuven Multidisciplinary Breast Centre | LMBC | Belgium | Commissie Medische Ethiek van de Universitaire Ziekenhuizen Kuleuven |
| Macedonian Breast Cancer Study | MABCS | Republic of North Macedonia | Ethic Subcommittee of Medicine, Pharmacy, Veterinary Medicine and Dentistry, Macedonia Academy of Sciences and Arts |
| Mammary Carcinoma Risk Factor Investigation | MARIE | Germany | Medizinische Fakultat Heidelberg Ethikkommission; Ethik-Kommission der Arztekammer Hamburg |
| Milan Breast Cancer Study Group | MBCSG | Italy | Comitato Etico Indipendente della Fondazione IRCCS "Istituto Nazionale dei Tumori" |
| Mayo Clinic Breast Cancer Study | MCBCS | USA | Mayo Clinic IRB |
| Multi-ethnic Cohort | MEC | USA | University of Southern California Health Sciences Campus IRB |
| Melanoma Inquiry of Southern Sweden | MISS | Sweden | Regional Ethical Board in South Sweden |
| Mayo Mammography Health Study | MMHS | USA | Mayo Clinic IRB |
| Memorial Sloan-Kettering Cancer Center | MSKCC | USA | Memorial Sloan-Kettering Cancer Center IRB/Privacy Board-B |
| Malaysian Breast Cancer Genetic Study | MYBRCA | Malaysia | University Malaya Medical Centre Medical Ethics Committee; Ramsay Sime Darby Independent Ethics Committee |
| Norwegian Breast Cancer Study | NBCS | Norway | Regionale Komitere for Medisinsk og Helsefaglig Forskningsetikk |
| Nashville Breast Health Study | NBHS | USA | Vanderbilt University Medical Center IRB |
| Northern California Breast Cancer Family Registry | NC-BCFR | USA | Stanford University IRB |
| North Carolina Breast Cancer Study | NCBCS | USA | Office of Human Research Ethics, the University of North Carolina, Chapel Hill |
| Nagano Breast Cancer Study | NGOBCS | Japan | Institutional review board of the National Cancer Center, Tokyo |
| Nurses Health Study | NHS | USA | Partners Human Research, Partners Healthcare System (PHS) IRB |
| Nurses Health Study 2 | NHS2 | USA | Partners Human Research, Partners Healthcare System (PHS) IRB |
| Leiden University Medical Centre Breast Cancer Study | ORIGO | Netherlands | Medical Ethical Committee and Board of Directors of the Leiden University Medical Center (LUMC) |
| NCI Polish Breast Cancer Study | PBCS | Poland | National Cancer Institute Special Studies Institutional Review Board (NCI-SSIRB) |
| Karolinska Mammography Project for Risk Prediction of Breast Cancer - Case-Control Study | pKARMA | Sweden | Regionala Etikprovningsnamnden i Stockholm (Regional Ethical Review Board in Stockholm) |
| The Prostate, Lung, Colorectal and Ovarian (PLCO) Cancer Screening Trial | PLCO | USA | National Cancer Institute Special Studies Institutional Review Board (NCI-SSIRB) |
| Prospective Study of Outcomes in Sporadic Versus Hereditary Breast Cancer | POSH | UK | South West Multi-centre Research Ethics Committee |
| Evaluation of Predictive Factors regarding the Effectivity of Aromatase Inhibitor Therapy | PREFACE | Germany | Friedrich-Alexander-Universitat Erlangen-Nurnberg Medizinische Fakultat Ethik-Commission |
| Predicting the Risk Of Cancer At Screening Study | PROCAS | UK | NRES Committee North West - Greater Manchester Central |
| Shanghai Breast Cancer Genetic Study | SBCGS | China | Vanderbilt University Medical Center IRB |
| Study of Epidemiology and Risk factors in Cancer Heredity | SEARCH | UK | Multi Centre Research Ethics Committee (MREC) |
| Seoul Breast Cancer Study | SEBCS | Korea | Seoul National University College of Medicine/Seoul National University Hospital IRB |
| Singapore Breast Cancer Cohort | SGBCC | Singapore | Cases: National Health Group (NHG) Domain Specific Review Board (DSRB); SingHealth Centralised Institutional Review Board (CIRB). Controls: National University of Singapore (NUS) IRB. |
| The Sister Study | SISTER | USA | Institutional Review Board of the National Institute of Environmental Health Sciences; NIH and the Copernicus Group Independent Review Board |
| Städtisches Klinikum Karlsruhe Deutsches Krebsforschungszentrum Study | SKKDKFZS | Germany | Medizinische Fakultat Heidelberg Ethikkommission |
| Swedish Mammography Cohort | SMC | Sweden | Regionala Etikprovningsnamnden i Stockholm (Regional Ethical Review Board in Stockholm) |
| Simultaneous Study of Gemcitabine-Docetaxel Combination adjuvant treatment | SUCCESSB | Germany | Ethikkommission der Medizinischen Fakultät der Ludwig-Maximilians-Universität München |
| Simultaneous Study of Docetaxel Based Anthracycline Free Adjuvant Treatment Evaluation | SUCCESSC | Germany | Ethikkommission der Medizinischen Fakultät der Heinrich Heine Universität Düsseldorf |
| Triple Negative Breast Cancer Consortium Study | TNBCC | Various | Mayo Clinic IRB |

##

## Web Resources

BCAC Consortium: <http://bcac.ccge.medschl.cam.ac.uk/>

ClinVar: <https://www.ncbi.nlm.nih.gov/clinvar/>

COSMIC: <https://cancer.sanger.ac.uk/cosmic>

Genetics Home Reference: <https://medlineplus.gov/genetics/>

MalaCards: <https://www.malacards.org/>

##

## Supplemental References

1. [Michailidou K, Lindström S, Dennis J, Beesley J, Hui S, Kar S, et al. Association analysis identifies 65 new breast cancer risk loci. Nature [Internet]. 2017 Nov 2;551(7678):92–4. Available from:](http://paperpile.com/b/EH80kH/yI6Dc) <http://dx.doi.org/10.1038/nature24284>

2. [Ionita-Laza I, McCallum K, Xu B, Buxbaum JD. A spectral approach integrating functional genomic annotations for coding and noncoding variants. Nat Genet [Internet]. 2016 Feb;48(2):214–20. Available from:](http://paperpile.com/b/EH80kH/FoKsD) <http://dx.doi.org/10.1038/ng.3477>
